# Spatiotemporal and hydrodynamic influences on microbial and exometabolite dynamics in coral reef and seagrass ecosystems

**DOI:** 10.1093/ismejo/wrag177

**Published:** 2026-07-06

**Authors:** Brianna M Garcia, Sharon L Grim, Yan Jia, Laura Weber, Cynthia C Becker, Mallory Kastner, Gretchen J Swarr, Melissa C Kido Soule, Aushaun Brown, Weifeng Zhang, Elizabeth B Kujawinski, Amy Apprill

**Affiliations:** Department of Marine Chemistry and Geochemistry, Woods Hole Oceanographic Institution, Woods Hole, MA 02543, United States; Department of Marine Chemistry and Geochemistry, Woods Hole Oceanographic Institution, Woods Hole, MA 02543, United States; Department of Applied Ocean Physics & Engineering, Woods Hole Oceanographic Institution, Woods Hole, MA 02543, United States; Department of Marine Chemistry and Geochemistry, Woods Hole Oceanographic Institution, Woods Hole, MA 02543, United States; Department of Marine Chemistry and Geochemistry, Woods Hole Oceanographic Institution, Woods Hole, MA 02543, United States; Department of Biology, Ithaca College, Ithaca, NY 14850, United States; Department of Marine Chemistry and Geochemistry, Woods Hole Oceanographic Institution, Woods Hole, MA 02543, United States; The MIT-WHOI Joint Program in Oceanography/Applied Ocean Science and Engineering, Cambridge 02139, and Woods Hole 02543, MA, United States; Department of Marine Chemistry and Geochemistry, Woods Hole Oceanographic Institution, Woods Hole, MA 02543, United States; Department of Marine Chemistry and Geochemistry, Woods Hole Oceanographic Institution, Woods Hole, MA 02543, United States; University of the Virgin Islands, 2 John Brewers Bay, St. Thomas, US Virgin Islands 00802; Department of Applied Ocean Physics & Engineering, Woods Hole Oceanographic Institution, Woods Hole, MA 02543, United States; Department of Marine Chemistry and Geochemistry, Woods Hole Oceanographic Institution, Woods Hole, MA 02543, United States; Department of Marine Chemistry and Geochemistry, Woods Hole Oceanographic Institution, Woods Hole, MA 02543, United States

**Keywords:** bacteria, archaea, microbial community, microbiome, metabolomics, dissolved organic matter, exometabolome, hydrodynamics, coral reef, seagrass

## Abstract

Coral reef and seagrass ecosystems provide critical storm protection and economic revenue to tropical coastal communities, and therefore, effective monitoring and restoration strategies are essential. Microorganisms, and the metabolites they produce and consume, are key drivers of coastal ecosystem function. However, microbially mediated metabolite recycling remains poorly understood, limiting its inclusion in conservation and restoration strategies. Here, we examine how seawater exometabolites and microorganisms vary in coastal ecosystems, across spatial and temporal scales and in relation to hydrodynamics. We characterized benthic seawater from two St. John, US Virgin Islands coral reefs (Yawzi and Tektite) and one seagrass meadow at dawn and mid-day over four consecutive days in January 2021. Using quantitative metabolomics and small subunit ribosomal RNA gene amplicon sequencing, we found that exometabolite and microbial community composition differed between sites. By applying hydrodynamic modeling, we determined that the daily changes and system variability were strongly influenced by water source origins. Mid-day offshore water intrusion at Yawzi reef likely drove exometabolite and microbial shifts toward oligotrophic taxa (e.g. SAR11, SAR86), whereas a high percentage of coastal source water in the seagrass site maintained stable exometabolite pools and supported diverse microorganisms. These findings demonstrate that geographically constrained site-level differences and hydrodynamics significantly impact exometabolite and microbial assemblages over short timescales. Integrating exometabolites, microorganisms, and hydrodynamics provides new insights into coastal ecosystem functioning useful for environmental monitoring and restoration strategies.

## Introduction

Tropical coastal ecosystems, including coral reefs and seagrass habitats, support the livelihoods of around a billion people worldwide through services such as storm protection, sediment stabilization, tourism, and carbon sequestration [1, 2]. These ecosystems are under significant and rapidly increasing threats due to climate change and human development [3–5]. Understanding and monitoring the health of coral reefs and seagrass habitats is important for informing conservation and remediation activities and for analyzing future risks facing coastal regions. Current monitoring programs predominantly use macroorganismal metrics, such as the diversity and abundance of stony corals, algae, and fish as sentinels of ecosystem health [6]. However, visual observations miss critical information about the microbial and chemical parameters that play into ecosystem health in a dynamic ocean [6, 7].

Marine microbes are primary producers and consumers of dissolved organic matter (DOM), form symbiotic and/or pathogenic relationships with corals and other macroorganisms, and have the potential for rapid growth and cellular turnover [8]. The most dynamic component of DOM is structurally diverse, low-molecular-weight molecules (exometabolites) that are rapidly transformed through microbial metabolism [8]. This tightly interconnected microbe–metabolite network can reflect real-time changes in environmental conditions, including nutrient and pollution levels, oxygen, temperature, and salinity [9–12]. Additionally, physical processes such as hydrodynamics can influence exometabolite and microbial dynamics within tropical coastal ecosystems. For example, reefs situated along the forereef are more exposed to offshore seawater and likely experience different inputs and turnover rates of exometabolites compared to reefs located in more protected bays. Understanding the influence of hydrodynamics on differing metabolite inputs can shed light on the tightly coupled responses of microbial growth and succession in seawater. Despite the importance of examining interactions between seawater exometabolites, microorganisms, and hydrodynamics, no studies have yet examined these three parameters simultaneously in tropical coastal environments.

Over the last decade, studies have established a strong foundation of knowledge regarding the taxonomic composition of microbial communities within tropical coastal ecosystems. Seawater microorganisms on shallow coral reefs include high standing stocks of photoautotrophic cells (e.g. *Prochlorococcus, Synechococcus*) and varying densities of heterotrophic bacterial taxa (oligotrophs: e.g. SAR11, SAR86, NOR5; and copiotrophs: e.g. *Flavobacteriaceae, Rhodobacteraceae*) [13]. Reef site and biogeography serve as the primary control on microbial composition [14–18], likely due to the combination of ocean and reef conditions. Additional controls include macro-organismal density and diversity [19–22], physicochemical conditions of the seawater [23–25], and disturbance events such as disease [26, 27]. For seagrasses, microbial composition is linked to site as well as tidal conditions and salinity [28–30]. The largest within-site impact on temporal dynamics within reefs and seagrass environments appears to be the nightly division of *Prochlorococcus* and *Synechococcus* cells [28, 31, 32]. Collectively, seawater microorganisms can serve as bioindicators of tropical coastal ecosystems and ocean conditions, and there is momentum to provide a more unified platform for monitoring programs [7, 33].

Compared to seawater microorganisms, much less is known about the exometabolome composition and dynamics in coral reef and seagrass habitats. Metabolomics studies have largely focused on the intracellular composition of coral tissues and seagrass leaves, roots, and rhizomes; relatively few studies have addressed the chemical composition and spatio-temporal dynamics of their exometabolomes [16, 34–36]. Like microbial communities, DOM composition varies with biogeography [16], disturbance [37], and anthropogenic and terrestrial influences [17, 38]. Coral reef primary producers release distinct chemicals with diurnal impacts on microbial communities [20] and water chemistry parameters, such as dissolved oxygen, pH, and nutrient availability [39]. Specific chemical classes differ on diel timescales based on the benthic producer, with coral and fleshy algae found to release more bioavailable lipids and organic nitrogen compounds at night and labile heterocyclic compounds during day [40]. Research on seagrass exometabolomes is more limited; however, studies indicate that eelgrass leaf exudates can contribute substantially to marine DOM, particularly with allelochemicals [35]. Quantitative metabolomic studies of coral reefs and seagrass meadows have been limited by methodological constraints. However, emerging approaches such as chemical derivatization support the quantification of new exometabolites, as well as the flux dynamics of individual compounds or compound classes [34, 41, 42].

Translating molecular-level insights into ecosystem-scale processes requires consideration of hydrodynamics and physical geography, because water flow strongly influences microbial activity and metabolite transfer [43–45]. Coastal circulation directly affects nutrient delivery, disease transport, contaminant distribution, and larval dispersal [45]. Numerical simulations of nutrient uptake in coral reef systems suggest a pattern of increased nutrient uptake near the reef crest, with decreasing nutrient concentrations downstream across the reef [43], underscoring that local flow patterns shape resource availability. Empirical studies support these patterns. For example, dissolved organic carbon and microbial biomass are depleted on fringing and barrier reef habitats in Mo’orea, French Polynesia, alongside differentiation of bacterioplankton communities between offshore, forereef, backreef, and bay environments [46]. Similarly, high biogeochemical similarity has been observed across forereefs in the Jardines de la Reina, Cuba reef system. It was hypothesized that regional-scale hydrodynamics, such as the Caribbean current, may homogenize exometabolite pools by flushing reefs with oligotrophic water [36]. Together, these examples illustrate that water flow can either concentrate or dilute microbial–metabolite interactions, yet the extent to which hydrodynamics mediate ecosystem-scale chemical diversity and microbial community composition remains poorly resolved. Addressing this gap requires systems where microbial, exometabolite, and hydrodynamic measurements can be directly integrated.

The coral reefs of southern St. John, US Virgin Islands, present an ideal framework to examine exometabolite–microbial–hydrodynamic patterns. Extensive studies over four decades provide a rich ecological context for understanding current community dynamics relative to well-documented coral loss and habitat change [47–50]. The microorganisms on these reefs and adjacent seagrass meadows have been studied for 10 years [18, 26, 28, 31] and the exometabolites recently explored [20, 34], establishing a baseline of microbial and chemical diversity. Further, a realistic high-resolution hydrodynamic model of the St. John coastal region was recently constructed, validated, and used to calculate the cross-bay variability of water residence time, a key factor influencing nutrient delivery, disease spread, larval dispersal, and contaminant transport [44, 45]. Building on this extensive historical and environmental knowledge, this study examined the composition and diversity of seawater microorganisms and exometabolites in a well-characterized tropical embayment in relation to relevant influences, including site, time of day, and water sources. Over four days, we collected benthic depth seawater at dawn and mid-day at two coral reefs and one seagrass habitat. Seawater was processed and analyzed for dissolved exometabolites using a benzoyl chloride (BC) derivatization approach and quantitative metabolomics [41], and for microbial community composition using small subunit (SSU) ribosomal RNA gene amplicon sequencing targeted to bacteria and archaea. Additionally, a hydrodynamic model previously established for this region and an associated particle-tracking model were used to trace back the sources of the water samples over the four sampling days. Our results revealed that site, hydrodynamics, and time of day (dawn vs. mid-day), all influenced the observed microbial–exometabolite patterns and serve as important drivers of exometabolite and microbial dynamics in coastal ecosystems.

## Materials and methods

### Sample collection

**Figure 1:**
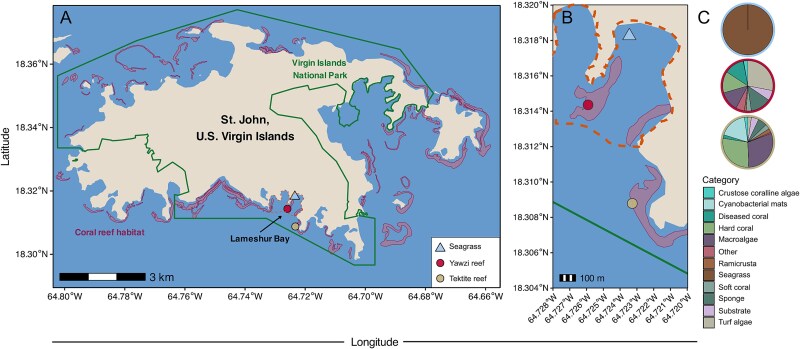
Map of St. John, US Virgin Islands showing (A) the Virgin Islands National Park (solid boundary) and surrounding coral reefs. Sampling sites are indicated for two coral reef habitats, Yawzi reef and tektite reef, and one seagrass meadow. The boundary of the larger Lameshur Bay region is shown (dashed boundary). (B) Enlarged view of sampling sites within the Great Lameshur Bay region. (C) Pie charts showing the average benthic composition across four transects at each site, as determined using visual point intercept surveys. Each color within the pie represents a surveyed category. Surrounding outline colors match the corresponding site color on the map. Map shapefiles were obtained from open-access sources: Stanford EarthWorks (island boundary), the National Park Service Land Resources Division (park boundary), and NOAA National Centers for Coastal Ocean Science (benthic habitat map).

Seawater samples were collected in January 2021 in St. John, US Virgin Islands (Table 1) within the Virgin Islands National Park (Fig. 1) at two coral reefs, Tektite (TK) and Yawzi (YZ), and one seagrass meadow (SG). Sampling locations (TK, YZ, and SG) were selected based on their proximity (~0.14 km^2^) within the Great Lameshur Bay region and their unique benthic compositions determined via point intercept benthic surveys (Fig. 1C) [34]. The seagrass site was composed of seagrass and surrounding sediment, whereas the two reefs differed in benthic composition (Fig. 1C) including their coral-to-algae ratio. The coral-to-algae ratio at TK (1.1 ± 0.4 se) was higher than at YZ (0.5 ± 0.1 se), indicating a greater relative dominance of coral over algae at TK, which we hypothesized would be reflected in the exometabolite and microbial community measurements. Samples were collected at ~6 am (dawn) and ~ 2 pm (mid-day) local time over four consecutive days. Scuba divers collected benthic seawater (within 0.25 m of the benthos) in acid-washed Niskin Bottles (General Oceanics, Miami, FL, USA) at three randomly selected locations at each site. Replicates within each site were all within 15 m of the Global Positioning System (GPS) coordinate and were centered over the representative habitat. After ascent, the Niskins were drained into individual polycarbonate (PC) bottles (Nalgene, Thermo Scientific, Waltham, MA, USA), placed on ice, and processed within 2 h of collection. Each individual Niskin bottle was considered a representative sample of each site (biological replicate, herein defined as a replicate), with a total of three replicates collected per site at each sampling timepoint.

**Table 1:** Sampling site characteristics and sampling numbers.

Site characteristics					Replicates (*n*) Exometabolome		Replicates (*n*) Microbiome	
Site	Latitude	Longitude	Average depth (m)	Benthic cover	Dawn	Mid-day	Dawn	Mid-day
Tektite	18.309°N	64.723°W	8.4	Higher coral:algae	12	13	12	12
Yawzi	18.314°N	64.726°W	8.5	Higher algae:coral	13	12	12	12
Seagrass	18.317°N	64.723°W	3.6	Seagrass dominated	12	12	12	12

### Total organic carbon and microbial abundances

From each sample, 40 ml of unfiltered benthic seawater, in combusted borosilicate glass vials (Thermo Scientific, Waltham, MA, USA), was acidified to pH 2 with 75 μl of concentrated phosphoric acid. Total organic carbon (TOC) and total nitrogen (TN) concentrations were analyzed using a Shimadzu TOC-L analyzer with a TNM-L module. Measurements were made using potassium hydrogen phthalate and potassium nitrate as standard solutions.

For the enumeration of *Prochlorococcus, Synechococcus*, picoeukaryotic cells, and unpigmented cells (generally heterotrophic bacteria and archaea), 1.4 ml of unfiltered seawater was fixed with 8% paraformaldehyde (1% final concentration), incubated for 20 min at 4°C in the dark, flash frozen in a dry shipper (MVE, Ball Ground, GA, USA), and then stored at −80°C prior to analysis. Samples were thawed and stained with Hoechst 34580 (1 μg/ml, final concentration) and analyzed using a Beckman-Coulter Altra Flow Cytometer (Brea, CA, USA) endowed with two argon ion lasers, tuned to ultraviolet (200 mW) and 488 nm (1 W) excitation wavelengths. Side and forward scatter, as well as fluorescence signals, were collected using the appropriate filters designated for Hoechst-bound DNA, phycoerythrin, and chlorophyll. FlowJo software (Tree Star, Inc.) was used to bin populations and estimate the abundances of *Prochlorococcus, Synechococcus*, picoeukaryotes, and unpigmented cells according to published protocols [51].

### Filtration for microbial and metabolomics sample processing

Benthic seawater was filtered through polytetrafluoroethylene 0.2 μm, 47 mm filters (Omnipore, MilliporeSigma, Burlington, MA, USA) in perfluoroalkoxy (PFA) in-line 47 mm filter holders (Advantec MFS, Inc., Dublin, CA, USA) using peristalsis (MasterFlex L/S pump and pump heads, Vernon Hills, IL, USA). Thermoplastic elastomer tubing and acid-washed fluorinated ethylene propylene tubing (890 Tubing, Nalgene, Thermo Scientific, Waltham, MA, USA) were used to pump seawater through the filter membrane and into acid-washed PC collection bottles (Nalgene, Thermo Scientific, Waltham, MA, USA). Filters with captured cells were placed in cryovials (Corning, Corning, NY, USA), and flash frozen in a dry shipper, where they remained stored until transport to Woods Hole Oceanographic Institution. Frozen filters were stored at −80°C until subsequent processing. The corresponding filtrate (40 ml) was collected into combusted amber vials (EP, Thermo Scientific, Waltham, MA, USA) for BC derivatization and targeted metabolomics measurements (described below). Exometabolomics samples were stored at −20°C and transported frozen using Techni Ice sheets.

### Exometabolite sampling processing

Filtered, frozen seawater samples were thawed and 25 ml from each sample was transferred into a combusted 40 ml amber glass vial (EP, Thermo Scientific, Waltham, MA, USA) for ^12^C-benzoyl chloride (^12^C-BC) derivatization conducted according to a previously published method [41]. A nine-point standard curve was derivatized (^12^C-BC) in parallel with samples, using 25 ml of off-reef seawater as the matrix and included the values: 0, 0.005, 0.01, 0.03, 0.07, 0.1, 0.3, 0.7, and 1 ng/ml. Stable isotopically labeled internal standards (^13^C-labeled SIL-IS) were derivatized in parallel, using ^13^C-BC, and spiked into all experimental and standard curve samples at a final concentration of 0.2 ng/ml. Derivatized samples and standards were then extracted by solid-phase extraction (SPE) using 6 ml, 1 g Bond Elut PPL cartridges (Agilent, Santa Clara, CA, USA), and the final eluent was dried in a Vacufuge Plus concentrator (Eppendorf, Hamburg, Germany) to near-dryness at 30°C. Dried samples were stored at −20°C until mass spectrometry analysis, at which time each sample was reconstituted in 100 μl of 5% acetonitrile (MeCN), transferred to a 2 ml LC-MS vial (SureSTART, Thermo Scientific, Waltham, MA, USA) with a small volume (350 μl) insert (SureSTART, Thermo Scientific, Waltham, MA, USA), and topped with 5 μl of 100% MeCN. Samples were stored at 4°C until instrumental analysis (Supplemental Methods). BC derivatization and SPE were conducted according to standard protocols described in detail elsewhere [34, 52–54].

### Microbiome sample processing

DNA was extracted from seawater filters using DNeasy PowerBiofilm Kits (Qiagen, Hilden, Germany) according to the manufacturer’s protocol, with a blank (control) per extraction round. The eluted DNA served as the template for barcoded polymerase chain reaction (PCR) to amplify the hypervariable 4 region (V4) of the SSU rRNA gene using the 515FY [55] and 806RB [56] primers, respectively. The selected primers are specialized to recover marine bacteria and archaea [55, 56]. The 50 μl reactions contained: 2 μl DNA template (1:10 diluted), 0.5 μl of GoTaq DNA Polymerase (hot start, 5 units/μl), 1 μl each of forward and reverse primers at 10 μM, 1 μl of 10 mM deoxynucleoside triphosphate mix, 5 μl of 7.5 mM MgCl_2_, 10 μl GoTaq 5× colorless flexi buffer (all reagents Promega), and 29.5 μl nuclease-free water. Genomic DNA from Microbial Mock Community B (Even, Low Concentration), v5.1L, for 16S rRNA Gene Sequencing, HM-782D, was used as our sequencing control. Nuclease-free PCR-grade water was our negative PCR control. PCR cycling conditions were as follows: 95°C for 2 min; 29, 30, 32, or 34 cycles of 95°C for 20 s; 55°C for 15 s; 72°C for 5 min; and finally, 72°C for 10 min, before a hold at 4°C. Cycle number varied to optimize amplification; specifically, we amplified each sample to the cycle number that produced a detectable band. DNA extraction kit controls were amplified at the highest PCR cycle number of any of the samples in each extraction batch. The barcoded PCR products were purified using the MinElute PCR Purification kit (Qiagen, Hilden, Germany) and their concentrations were measured using Qubit 2.0 fluorometry. Purified PCR products were diluted to 1 ng/μl and pooled. Samples were sequenced 2 × 250bp on a MiSeq System (Illumina) at the Roy J. Carver Biotechnology Center of the University of Illinois at Urbana-Champaign.

### Exometabolite data processing

Thermo .raw files were processed using Skyline (v. 21.2.0.568) [57, 58]. Peaks corresponding to detected metabolites were integrated based on accurate mass (±10 ppm), retention time, and MS/MS fragment ion confirmation. Calibration curves for each compound were constructed based on the metabolite standard concentration (ng/ml) versus the integrated light-to-heavy peak area ratios. Exported quantification tables from Skyline containing peak areas for each metabolite were imported into MATLAB (v. R2022a). An in-house MATLAB script (considerSkyline.m) was used to generate linear regression calibration curves, quantify each metabolite, calculate limits of detection (LOD) and quantification (LOQ), and merge positive and negative polarity mode data [59]. The resulting merged quantification table was further filtered based on defined quality assurance and quality control (QA/QC) metrics (Supplemental Methods). The resulting quantification table containing nanomolar (nM) concentrations was log_2_(*x* + 1)-transformed for downstream statistical analyses to handle the dynamic range of metabolite concentrations appropriately and avoid negative transformed values due to imputation of values less than the LOD.

### 16S rRNA gene sequencing data processing

Amplicon libraries were analyzed using the DADA2 (v. 1.26.0) package in R [60]. Read pairs were assessed for quality, trimmed of primers, and used to estimate sequencing error rates. Read pairs were merged to cover the length of the 16S rRNA gene V4 region and the resulting amplicon sequence variants (ASVs) were checked for chimeras. Taxonomy was assigned to ASVs using the Silva v.138.1 database [61]. After removing sequences assigned to potential contaminants (*n* = 7617; Supplemental Methods) and of eukaryotic origin (chloroplasts, mitochondria, and eukaryotes: *n* = 171 607), the dataset of 72 samples retained 6 136 788 sequences representing 10 274 ASVs. ASV sequence counts were per-sample-normalized to relative abundance, after comparing various data transformations (centered log-ratio, robust centered log-ratio, relative abundance; Table S13 and Supplemental Results). For specific data processing parameters and details on taxonomic assignments, see the Supplemental Methods.

### Statistical analyses

Statistical analyses for both exometabolite and microbiome data are briefly described herein. Detailed methods, parameters, and results can be found in the Supplemental Methods and tables. All statistical analyses were conducted in R (v4.2–4.3) within RStudio. Exometabolite concentrations were non-normal (Shapiro–Wilk *P*-value <.05); therefore, nonparametric approaches were used throughout. Community structure was characterized using dissimilarity-based methods, with Bray–Curtis dissimilarity applied to exometabolite profiles and Morisita–Horn dissimilarity to amplicon relative abundances, and visualized using non-metric multidimensional scaling (NMDS) (both global and site-specific ordinations). Differences in beta diversity (Δβ) and within-group dispersion were assessed using multivariate dispersion metrics and nonparametric Kruskal–Wallis tests or bootstrapped *t*-tests. The effects of site, sampling time, and sampling day were tested using adonis2 implementation of permutational multivariate analysis of variance (PERMANOVA), with interaction terms included and pooled where necessary to avoid negative variance estimates. Relationships with environmental and biological predictors were further evaluated using distance-based redundancy analysis. Univariate differences in exometabolite concentrations across groups were assessed using Kruskal–Wallis tests with pairwise Wilcoxon comparisons, and multiple testing was controlled using the Benjamini–Hochberg false discovery rate (*q* < 0.05). Differentially abundant microbial taxa were identified using complementary approaches (corncob and DESeq2). To be most effective at culling putative false positives that arose from multiple tests of nonindependent observations, significant taxa were identified from each approach by modeling the local false discovery rates (LFDRs). Raw *P*-values were used to model 1%, 5%, and 10% LFDR quantiles. After examining the *P*-values distributed in those quantiles, the corresponding adjusted *P*-values, modeled estimates, and coefficients for all observed taxa, an experiment-wise error rate of 5% (α = 0.05) and 10% (α = 0.10) was determined for corncob and DESeq2, respectively. Across both methods, 181 unique ASVs were flagged to be significantly differentially abundant in paired sample groups, which we retained for further analyses. Finally, site-specific associations between exometabolites log2(*x* + 1) concentrations and microbial taxa relative abundances were assessed using Spearman correlations. Spearman’s rho (ρ) of |0.5| and *P*-values corresponding to a 1% FDR were considered significant (Table S12).

### Hydrodynamic modeling

We used an offline particle tracking model, ROMSPath [62], to identify the origins of the exometabolite and microbiome water samples that were collected. The 3D hourly velocity field from the hydrodynamic model 2021 simulation (Supplemental Methods) was used in ROMSPath to calculate the backward particle trajectories with a time step of 60 s. Note, that “particle” in this context means virtual particles used in the hydrodynamic model to track the movement of the water, and “particle-tracking model” is a specific physical oceanography term. Results of the model hindcast simulation in 2016–22 were validated against historical observations on the St. John coastal region. A total of 24 particle-tracking backward simulations were carried out per water sample. In each simulation, model particles were released every 10 min for a 2-h window centered at a sampling time and then tracked for 24 h to capture day-to-day and diurnal influences on particle trajectories. The released model particles were evenly spaced every 2 m horizontally across a 50 m × 40 m area centered on each sampling station and every 1 m vertically over the whole water column. The number of model particles within the larger Lameshur Bay region at each time was counted. The larger Lameshur Bay region was selected to represent a microbial environment distinct from the open coastal ocean. This boundary was chosen to focus our study on sites under stronger coastal influence, which are known to harbor characteristic microbial communities [26]. Additionally, defining this region constrained our analysis to the Virgin Islands National Park, excluding reef sites outside this boundary that have previously been shown to possess unique exometabolome and microbiome profiles [28, 34]. Because of the high spatial density of released model particles, the percentage of particles originating from the larger Lameshur Bay region at each time point is considered representative of the proportion of water samples derived from this area. A linear model (stats::lm) and linear mixed model (via lme4::lmer) were used to determine the influence of the water source on the exometabolome and microbiome variability using average beta-dispersion distances previously calculated for each site. Given that the dawn and mid-day sampling times were separated by ~7 h, the average percent of modeled particles within the greater Lameshur Bay region was calculated at each site 7 h before sampling across the four sampling days. Linear regressions were constructed based on the linear model outputs. Site was included as a random effect in the linear mixed model.

## Results

### Lameshur Bay coral reefs and seagrass meadow harbor distinct pools of polar exometabolites and microbial communities

**Figure 2:**
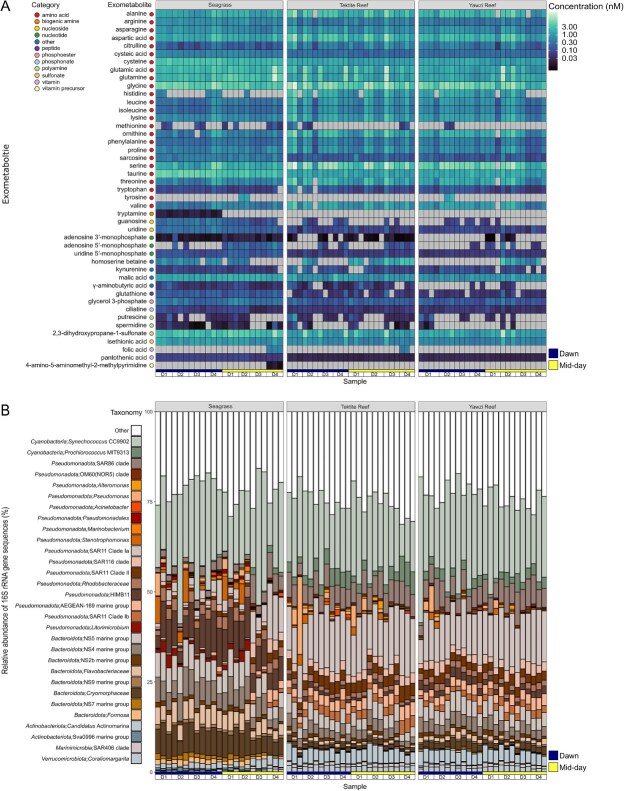
Exometabolites and microbes in coastal seawater sites. (A) Heatmap displaying the concentration (nM) of quantified exometabolites (*y*-axis) in seagrass, Yawzi reef, and Tektite reef (*n* = 3–4). Each column represents an individual sample. Samples are organized along the *x*-axis by sampling day (denoted using “D#” to represent sampling Day 1–4) within a given sampling time. Sampling times are color-coded at the bottom of each facet. Concentrations are shown using a log-scaled color gradient to facilitate visualization across the wide dynamic range. Concentrations that fell below the limit of detection are represented by gray-filled boxes. Exometabolites (*y*-axis) have been ordered based on metabolite class with their respective categories color-coded and displayed as filled circles next to each metabolite name. Sampling times are color-coded at the bottom of each facet. (B) Taxonomy bar chart displaying the relative abundance (*x*-axis) of the 30 dominant lineages (as specific as genus) observed in each sampling site: seagrass, Yawzi reef, and Tektite reef. Taxonomic groups beyond the top 30 are grouped into the “Other” category. Each bar represents an individual sample. Samples are organized along the *x*-axis by sampling day within a given sampling time. Sampling times are similarly color-coded at the bottom of each facet.

We quantified 45 amine- and alcohol-containing exometabolites with concentrations spanning roughly four orders of magnitude, ranging from 5 picomolar (pM) to 30 nanomolar (nM; Table S1). All 45 exometabolites were quantified in the seagrass meadow, whereas 42 were quantified at both reefs (Fig. 2A). For the seawater microbial community, an average of 87 355 sequences (range of 13 915–114 423; similar sequencing effort between sites and sampling times, *P*-value = .43 via Kruskal–Wallis) and 169–827 ASVs per sample were recovered (Table S2). Of the distinctive taxa identified, 30 ASVs were responsible for at least 82.4% of the sequences per sample (Fig. 2B, Table S3), representing consistency across samples.

**Figure 3:**
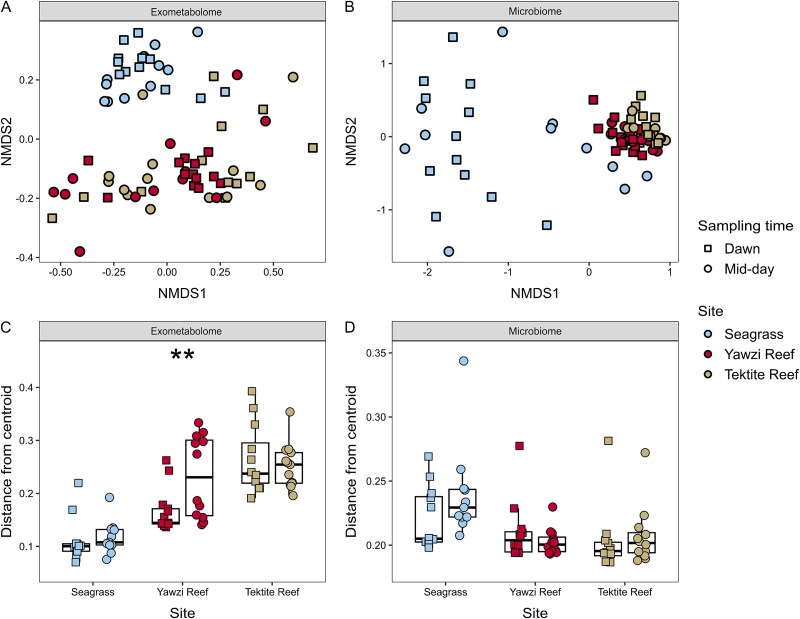
Coral reefs and seagrass site harbor distinctive exometabolomes and microbiomes. NMDS of microbiomes from seagrass, Yawzi reef, and Tektite reef. (A) Bray–Curtis dissimilarities between samples’ exometabolite concentrations were used to generate the NMDS. (B) Morisita–Horn dissimilarities between samples’ ASV relative abundance profiles were used to generate the NMDS. PERMANOVA results are presented in Table 2. (C) Box-and-whisker plot showing beta-dispersion distances for the exometabolome data within each site and sampling time. Each point represents the difference in Bray–Curtis dissimilarity of a sample from the median dissimilarity (50th percentile line) for the time- and site-specific group. Intra-site and inter-site significant differences in dissimilarity distances are indicated with “^**^” (pairwise *t-*test *P*-value <.05). (D) Box-and-whisker plot showing beta-dispersion distances for the 16S rRNA gene sequencing data within each site and sampling time. Each point represents a sample’s difference in Morisita–Horn dissimilarity from the median dissimilarity (50th percentile line) for the time- and site-specific group.

An unsupervised NMDS approach showed that exometabolite and microbial profiles were site-specific with the two reef sites clustered together and separate from the seagrass, irrespective of sampling time (Fig. 3A and B). Clustering patterns by NMDS were similar for the exometabolome and microbiome. In contrast, the samples’ beta diversity distributions (dispersion) were visibly and quantitatively different within the two ordination plots (Fig. 3C and D). A PERMANOVA analysis identified the significant factors (*P*-value <.05) driving the exometabolome and microbiome variability observed in the NMDS plot (Table 2); site was the largest significant factor structuring both the exometabolome and microbiome, explaining 31.3% and 47.3% of the variability, respectively. Exometabolome and microbiome profiles of Tektite and Yawzi reefs had significantly different diversity distributions (bootstrapped pairwise *t*-tests, exometabolome *t* = 4.97, microbiome *t* = 2.93, *P*-value <.01), and the seagrass site was significantly different from each reef site (exometabolome *t* = −7.97 to −13.9, microbiome *t* = 6.50–9.40, *P*-value <.001, Table S4).

**Table 2:** PERMANOVA and RDA results for exometabolome and microbiome.

	Exometabolomes				Microbiomes			
Source	Df	Pseudo *F*	*P*-value	% of variation explained in model	Df	Pseudo *F*	*P*-value	% of variation explained in model
Site	2	15.6	**.0001**	31.3	2	78.3	**.0001**	47.3
Time	1	4.0	**.0200**	15.8	1	15.7	**.0002**	21.2
Day	3	1.3	.2500	9.1	3	7.6	**.0007**	14.7
Day × Site	6	2.2	**.0100**	11.7	6	2.4	**.0390**	8.3
Time × Day	3	1.3	.2700	8.9	11	0.3	.9500	3.1
Time × Site	2	1.1	.3500	8.4				
Time × Day × Site	6	0.8	.7300	6.9				
Residual	49			7.9	47			5.3
Total	72				70			

Of the 45 quantified exometabolites, 34 showed significantly differentially abundant (SDA) concentrations between sites, within each sampling time (dawn or mid-day). Specifically, 32 exometabolites differed significantly at dawn and 29 at mid-day (false discovery rate [FDR]–adjusted *P*-value <.05, Pairwise Wilcoxon Rank Sum Test; Table S6). Exometabolite concentrations were largely elevated in the seagrass site, with only 11 exometabolite concentrations significantly higher at either Yawzi or Tektite reef relative to the seagrass. The seagrass exometabolome was generally characterized by elevated concentrations of nucleotides (e.g. 3′AMP, 5′UMP), organosulfur compounds (e.g. DHPS, taurine), and stress-protective osmolytes (e.g. homoserine betaine; Figs 2A and S3). In contrast, both coral reef exometabolomes were dominated by proteinogenic amino acids such as isoleucine, leucine, phenylalanine, serine, and threonine (Figs 2A and S3). Pair-wise exometabolite differences between the two reef sites were subtle, with only three SDA exometabolites (3′AMP, γ-aminobutyric acid (GABA), and isethionic acid). These results suggest that the seagrass meadow we examined differed significantly in composition from proximal reefs.

A similar pattern was observed in the microbiome. Across all three sites, 181 ASVs differed significantly in relative abundance based on concordance between corncob (5% FDR, *P*-value <.054) and DESeq2 (10% FDR, *P*-value <.050, Table S7, Table 3). The two reef sites were enriched in cyanobacteria (i.e. *Prochlorococcus, Synechococcus*), oligotrophic heterotrophs such as *Candidatus* Actinomarina, and SAR11 (Figs 2B and S3). The seagrass site, in contrast, showed higher abundances of heterotrophic bacteria including *Rhodobacteraceae*, multiple Bacteroidia lineages (NS4, NS5, NS9), and *Cryomorphaceae* (Figs 2B and S3). These patterns aligned with cell counts, which showed that *Synechococcus* populations were two to three times more abundant than *Prochlorococcus* overall, and consistently elevated in the reef sites relative to the seagrass site (Fig. S1b, Table S8).

**Table 3:** Frequency of significantly differentially abundant (SDA) ASVs and exometabolites across comparisons.

		Number of SDA ASVs	Average relative abundances of SDA ASVs (%)			Number of SDA exometabolites
			Min	Median	Max	
**Temporal**						
Dawn vs. mid-day	Seagrass	5	0.24	0.81	3.30	11
	Yawzi	30	0.00	0.26	2.41	6
	Tektite	0	NA	NA	NA	3
**Spatial**						
Dawn	Seagrass vs. Yawzi	140	0.00	0.13	7.15	32
	Seagrass vs. Tektite	122	0.00	0.15	7.18	26
	Yawzi vs. Tektite	27	0.01	0.28	3.75	3
Mid-day	Seagrass vs. Yawzi	119	0.00	0.16	7.65	21
	Seagrass vs. Tektite	95	0.00	0.20	7.65	25
	Yawzi vs. Tektite	2	0.72	0.95	1.47	1

### Fine-scale temporal dynamics of the microbiome and exometabolome

Sampling time was a large contributor to microbiome and exometabolome differences between samples, explaining >15% of the observed variability (Table 2). Site-specific NMDS ordinations with 95% confidence intervals showed little separation by time for either the exometabolome or microbiome (Fig. S2a and b), with the largest temporal separation observed in the seagrass exometabolome, although confidence intervals of the two groups overlapped. However, diel changes in the exometabolomes and microbiomes showed significantly different dissimilarity patterns (Table S4). We calculated the mean difference in beta diversity, here reported as Δ$\beta$, from groups of sample beta diversity values to quantify whether communities became more or less dissimilar between dawn and mid-day. In the seagrass, microbial diversity significantly increased at mid-day (Δ$\beta$ = +0.064, *t* = −4.27, *P*-value <.001), and exometabolome diversity did not change (Δ$\beta$ = +0.005). No significant temporal shifts were detected in either microbiome (Δ$\beta$ = 0.015) or exometabolome diversity (Δ$\beta$ = −0.028) at Tektite reef. At Yawzi reef, microbial diversity was significantly lower at mid-day (Δ$\beta$ = −0.031, *t* = 3.09, *P*-value = .003), whereas exometabolome diversity was significantly higher at mid-day (Δ$\beta$ = +0.125, *t* = −6.72, *P-*value <.001), highlighting a unique pattern of opposing dynamics between microbial and exometabolite communities at this site.

Beta-diversity dispersion was used to quantify the spread of samples within each time point (Fig. 3C and D, Table S5), providing a measure of how variable or homogeneous samples were relative to each group’s centroid. In the seagrass-associated communities, there was less dispersion in the exometabolome samples and relatively more dispersion in the corresponding microbiomes. The opposite pattern emerged for both coral reef sites (Fig. 3C and D). For the exometabolome, beta-dispersion (here represented as ${\sigma}_{\beta}^2$) increased from dawn to mid-day and was roughly two-fold higher in the examined reef sites compared to the seagrass. Although exometabolomes across all sites, as well as the microbiomes of Tektite reef and the seagrass site, showed this general increase in heterogeneity from dawn to mid-day, the increase was only significant at Yawzi reef (*P*-value <.05; median dawn ${\sigma}_{\beta}^2$ = 0.144; median mid-day ${\sigma}_{\beta}^2$ = 0.230) (Fig. 3C). In contrast, Yawzi reef microbiome beta dispersion values were generally lower at mid-day compared to dawn, although not significantly (median dawn ${\sigma}_{\beta}^2$ = 0.204; median mid-day ${\sigma}_{\beta}^2$ = 0.200) (Fig. 3D).

**Figure 4:**
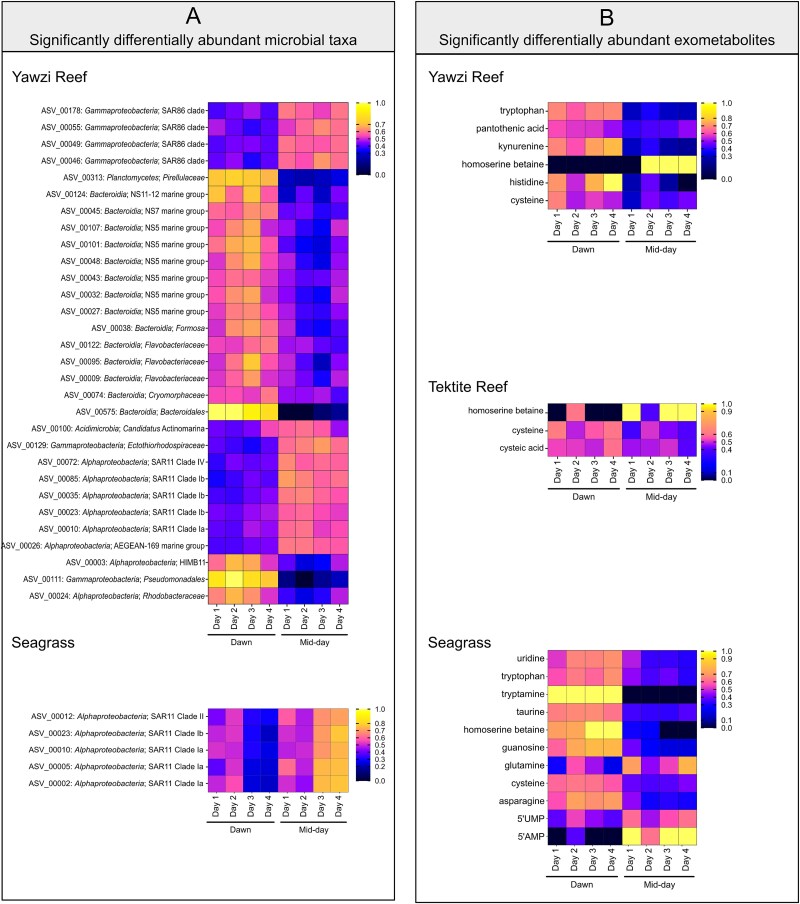
Temporally significant ASVs (A) and exometabolites (B) in Tektite reef, Yawzi reef, and Seagrass seawater. Thirty-three unique ASVs were considered temporally significant based on concordance between corncob (5% FDR, *P*-value <.054) and DESeq2 (10% FDR, *P*-value <.050). Fifteen unique exometabolites were considered significant based on a Kruskal–Wallis test with FDR-adjusted *P*-value <.05. For each ASV and exometabolite, the median value across replicates at each time point and sampling day was calculated and then normalized to that day by dividing by that feature’s total daily abundance or concentration. Warmer colors indicate higher normalized abundance, and cooler colors indicate lower normalized abundance.

Diel variation in exometabolite concentrations and microbial ASV relative abundances was observed across the three sites (Table 3); however, patterns varied in magnitude and direction (Fig. 4). Across all sites, 15 exometabolites and 33 ASVs showed significant temporal trends (Tables S6 and S7). The seagrass exometabolome had the largest number of SDA exometabolites (11) followed by Tektite (6) and Yawzi reef (3). Among the 15 significantly temporal exometabolites, cysteine and homoserine betaine were the only exometabolites found to exhibit significantly diel trends at all three sites. Additionally, homoserine betaine was the only exometabolite to show differing trends between sites (i.e. homoserine betaine was significantly elevated at dawn in the seagrass but was elevated at mid-day at both Tektite and Yawzi reefs). In contrast, cysteine concentrations were consistently higher at dawn compared to mid-day across all three sites and significantly elevated in the seagrass compared to either coral reef site.

In contrast to exometabolome temporal dynamics, which were strongest in the seagrass and minimal at the two coral reefs, microbial variation was more pronounced at Yawzi reef. The seagrass site showed limited microbial variation with time, with only five SAR11 ASVs, spanning clades Ia, Ib, and II, enriched at mid-day. At Tektite reef, no ASVs showed diel trends. At Yawzi reef, 30 ASVs varied significantly with time of day, including representatives of SAR11, SAR86, *Rhodobacteraceae, Flavobacteriaceae*, and other taxa. Only a small subset of SAR11 ASVs with significant temporal trends were observed at more than one site, highlighting a limited but recurring component of the microbial community that tracks diel changes.

### Hydrodynamics uniquely influence exometabolome and microbiome variability

**Figure 5:**
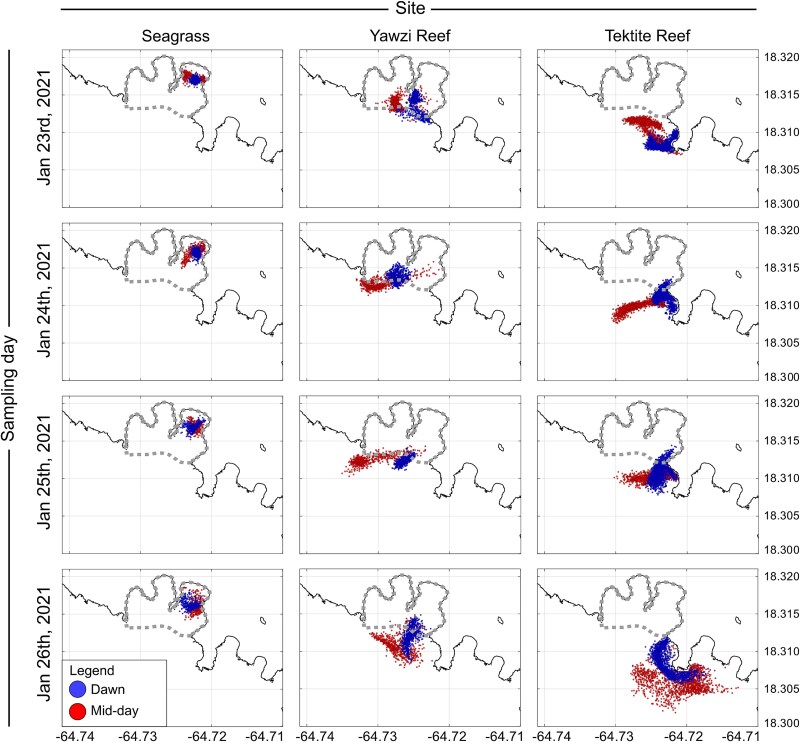
Hydrodynamics influence model particle movement on diel timescales. Hydrodynamic and particle-tracking models were used to trace the backward movement of benthic water 7 h before exometabolome and microbiome sampling times. Model particles were “released” between 5:00 and 7:00 a.m. LST (dawn) and between 12:00 and 2:00 p.m. LST (mid-day) in 10-mi intervals. Particle-tracking simulations were carried out for each of the 4 days sampled in this study. Positions of the model particles at 7 h before the sampling times at dawn and mid-day are shown by their respective latitude (*y*-axis) and longitude (*x*-axis) placements within the map. The boundary (dashed boundary) represents the larger Lameshur Bay region.

Particle-tracking simulations were used to identify water origins and estimate the time evolution of the percentage of the water samples originating from the larger Lameshur Bay region (Fig. 5). Water source percentage in the seagrass, located within the bayhead, was highly stable with little to no exchange outside of the bay up to 15 h before sampling each day (Fig. S4). Conversely, at Tektite reef the source water mainly originated from outside Lameshur Bay, with <20% originating from within Lameshur Bay up to 24 h prior to sampling (Fig. S5), consistent with expectations given Tektite reef’s location at the southeast corner of the larger Lameshur Bay region. Unlike the seagrass site and Tektite reef, particle-tracking simulations at Yawzi reef showed significantly different water sources at dawn vs. mid-day, with water origins outside Lameshur Bay observed as early as 1 h before sampling (Fig. S6). A higher percentage of Yawzi reef water, on average, was from the larger Lameshur Bay region at dawn (43.4%) compared to mid-day (33.3%).

Using a linear mixed model (Table S9), we found the planktonic microbiome was strongly associated with the proportion of water from Lameshur Bay (regression coefficient [*b*] = 0.00022 ± 0.00008 SE, *t* = 2.75, *R*^2^ marginal = 0.34) in addition to a strong site influence (*R*^2^ conditional = 0.41). Additionally, beta-dispersion was positively associated with the proportion of water from Lameshur Bay in both the linear regression (slope = +0.00024, adj. *R*^2^ = 0.37, Fig. S7b) and the linear mixed model (slope = +0.00022), indicating that water origin contributed meaningfully to microbial variability even after accounting for site-level differences. In contrast, the proportion of water from Lameshur Bay did not influence the exometabolome dispersion (*b* = 0.00056 ± 0.00047 SE, *t* = 1.20, *R*^2^ marginal = 0.05), which was strongly influenced by site (*R*^2^ conditional = 0.89). A linear regression of beta-dispersion against the proportion of water from Lameshur Bay suggested a negative relationship for the exometabolome (slope = −0.0012, adj. *R*^2^ = 0.54, Fig. S7a). However, when accounting for site as a random effect in a linear mixed model, this relationship was no longer evident (slope = +0.00056), indicating that site-level environmental structure, rather than water origin, drove the observed exometabolome patterns.

### Relationships between exometabolites and microbes reflect ecosystem dynamics

To link changes in log_2_(x + 1)-transformed exometabolite concentrations and the relative abundances of microorganisms, site-level Spearman correlations were conducted. In-depth investigations were conducted into microbial sequencing data transformations and are described in detail in the Supplemental Results. To identify more robust correlations, results were filtered using a Spearman’s rho cut-off of |0.5|, Benjamini–Hochberg adjustment to *P*-values <.05, and an FDR *q*-value of 1%. A total of 1704 significant correlations were detected (Table S10). Specifically, 980, 442, and 373 significant correlations were detected within the seagrass, Tektite, and Yawzi sites, respectively, between 42 exometabolites and 517 ASVs. Although cyanobacteria represent on average 20% or more of the bacterial community (Table S3), only 15 cyanobacterial ASVs were responsible for 49 unique correlations with 22 different exometabolites. *Synechococcus* and *Prochlorococcus* ASVs were involved in 30 and 2 significant correlations, respectively, while these exometabolites were associated with eight or fewer ASVs. In comparison, 21 of 517 ASVs had significant correlations with 10 or more exometabolites (Table S11), 3 of which were observed in more than one site, highlighting a high degree of specificity at the ASV level. Approximately 33% of the significant ASV–exometabolite correlations in the seagrass samples were unique to this site, whereas in both Yawzi and Tektite, 51% of correlations were observed only in each reef site. Surprisingly, the taxonomy of these multi-associated ASVs did not include typical photoautotrophic taxa. In contrast to the ASVs, individual exometabolites displayed greater redundancy, with pantothenic acid (vitamin B5) showing the highest number of correlations with ASVs (216), while tryptamine had the fewest (9). Homoserine betaine, which had significant diel patterns in all three sites, had correlations to 96 unique ASVs, including a strong positive correlation with a *Pelagibacter* (SAR11 Clade Ia) ASV in Yawzi Reef. Strong correlations (defined as rho > |0.9|) were observed for 18 unique ASVs responsible for 19 ASV–exometabolite relationships, 16 of which were found at Yawzi reef (Fig. S8).

## Discussion

By integrating quantitative exometabolomics, microbial community composition analysis, and hydrodynamic simulations over a short time series, our results reflect patterns and potential drivers of exometabolome and microbial dynamics in Lameshur Bay. Specifically, patterns differed among sites and across diel timescales and hydrodynamic conditions, highlighting the complexity of interactions within coastal ecosystems and the need for interdisciplinary approaches to disentangle the various physical, spatial, and temporal factors that collectively shape the function of these biodiverse environments.

The microbial communities of the two examined coral reefs were fairly homogeneous and characterized by taxa that are typically present in oligotrophic environments, including *Prochlorococcus, Synechococcus,* and SAR11. In contrast, the examined seagrass meadow displayed a more diverse microbial community enriched with the phylum Bacteroidota, including *Flavobacteriaceae, Rhodobacteraceae, Cryomorphaceae*, and *Halieaceae*, which have previously been identified as core microbial taxa overlying seagrass [63]. Additionally, sulfate-reducing bacteria *Desulfocapsaceae*, known to be associated with the seagrass rhizosphere [64–66], were enriched in the seagrass water. The consistent enrichment of these metabolically versatile groups suggests that the seagrass meadow represented a more organic-rich, dynamic habitat, supporting greater microbial diversity and functional potential than the adjacent reefs. Prior work in Lameshur Bay showed similar bulk nutrients between the investigated coral reef sites and seagrass meadow over diurnal and tidal scales [28]. In contrast, the mangrove forest that lies adjacent to the seagrass meadow was found to have significantly higher nutrient content and higher relative abundances of heterotrophic bacteria and archaea and picoeukaryotes [28]. The mangroves, although not measured in this study, could serve as an input into the Lameshur Bay seagrass meadow; however, additional studies looking at this relationship are needed. Our results indicate that reef and seagrass microbial communities within Lameshur Bay could be shaped by differences in habitat (seagrass vs. coral reef), reflecting distinct benthic composition and ecological functions rather than short-term diel or day-to-day fluctuations. Further, enhanced replication of coral reef and seagrass sites will inform the role of the ecosystem characteristics in these trends.

Although discernible pairwise site-level differences between the two coral reefs and seagrass microbial communities were observed, only minor differences existed between the two reefs. Microbial community composition between the reefs differed more at dawn than at mid-day, with only two significant ASVs, *Alphaproteobacteria* HIMB11 and Bacteroidia *Flavobacteriaceae,* detected at mid-day—both elevated at Yawzi reef. This consistency observed across adjacent reefs and over the span of multiple days suggests a largely uniform microbial community present among reefs within Lameshur Bay, consistent with prior findings [28]. A seven-year timeseries in this region found disturbances, such as disease outbreaks and hurricanes, played a major role in restructuring the microbial community composition on St. John reefs [26], further highlighting the need to understand these driving factors and how they change on both short and long-term timescales.

Site also served as the strongest driver of exometabolome composition. Whereas average bulk total organic carbon measurements were comparable across habitats (Fig. S1a), clear differences were observed in the concentration of individual amine- and alcohol-containing exometabolites, important labile components of DOM [8]. Seagrass exometabolomes were enriched in nucleotides, organosulfur compounds, and stress-protective osmolytes, whereas coral reef exometabolomes were dominated by proteinogenic amino acids (Fig. 2A, Fig. S3). Corals have been shown to synthesize [67] and release dissolved free amino acids into the water column, via their mucus [68] or as dissolved exudates as a function of photosynthesis [69], at higher abundances than other benthic reef constituents [70]. The abundance of organosulfur compounds in the seagrass exometabolome could reflect metabolic strategies used by the seagrass plant to transform and release excess sulfide into organic forms, thereby allowing the plants to survive in sulfide-rich and anoxic sediments [71]. Experiments are needed to confirm this hypothesis. Overall, the majority of exometabolite concentrations were elevated in the seagrass habitat compared to the two coral reefs. We hypothesize that this concentration effect in the seagrass reflected the more stable hydrodynamics of the meadow, allowing for exometabolites to accumulate over time. Comparatively at the two reefs, a measurable shift in source water had the potential to both bring in new metabolite producers and consumers and/or dilute exometabolite signals through physical mechanisms such as transport and mixing.

Diel and tidal cycles shape the rhythm of coastal ecosystems, with fluctuations in light and water movement jointly regulating the activity, distribution, and interactions of marine microorganisms. In this study, the seagrass site showed highly stable hydrodynamics over a 24-h time period, with little water movement into or out of Lameshur Bay. This static environment resulted in strong correlations between exometabolites and microbial ASVs (Supplemental Results), as well as the most temporally significant exometabolites, including amino acids and their derivatives (e.g. tryptophan, glutamine, cysteine), nucleosides and nucleotides (e.g. uridine, guanosine, 5′-AMP, 5′-UMP), and osmoprotective compounds (e.g. taurine, homoserine betaine). In contrast, SAR11 (clades Ia, Ib, and II) was the only microbe to show significant temporal fluctuations between dawn and mid-day. SAR11 was consistently elevated at mid-day in the seagrass, consistent with prior observations where SAR11 abundances were increased in the evening compared to the morning or afternoon [72]. Among the two coral reefs, homogenous exometabolome and microbiome profiles were observed at each distinct sampling time but with varying temporal dynamics and significantly fewer correlations compared to the seagrass environment. Specifically, more pronounced temporal changes were observed at Yawzi compared to Tektite. Yawzi also exhibited the unique trend where exometabolite profiles were significantly more diverse at mid-day, during the photosynthetically active diel period, whereas the microbial community, conversely, was less diverse.

Linking the exometabolome and microbiome via correlative analyses, while unable to definitively link specific microbes and metabolites via enzymatic pathways, integrates site- and/or habitat-specific factors that may influence their distributions without requiring explicit handling of those unknown parameters. We observed several exometabolites that were associated with multiple ASVs representing distinct taxonomic lineages, with a greater fraction being unique to either reef site (51% of ASV–exometabolite correlations) compared to the seagrass meadow (33%). Exometabolite homoserine betaine exhibited diel trends in every site and was elevated in the reef sites compared to the seagrass, but its presence in Yawzi reef was strongly and significantly associated with *Pelagibacter* (formerly known as SAR11 clade Ia) ASV_00068. Between the three sites, *Pelagibacter* ASV_00068 was significantly differentially less abundant in the seagrass at dawn compared to either reef site, as well as significantly more abundant in Yawzi reef at mid-day (Table S7a). Generally, five *Pelagibacter* ASVs were more abundant in the reef sites than in the seagrass site (Fig. S3), and one of these was also significantly more abundant at mid-day than dawn in both Yawzi reef and the seagrass site (Fig. 4). In other marine systems, the cyanobacterial osmolyte glycine betaine links SAR11 and *Prochlorococcus* [73]. SAR11 have notably small genomes among free-living bacteria, relying on enzyme multifunctionality to succeed in dynamic environments. Culture-based studies have confirmed the multifunctionality of *Pelagibacter’s* glycine betaine transporter to allow for transport of structurally similar compounds [74]. Homoserine betaine, an osmolyte first characterized in *Trichodesmium* [75], a bloom-forming, filamentous, ubiquitous cyanobacterium, was not directly tested in this study for microbial uptake, but the strong correlations detected here lead one to hypothesize that this molecule could meet these criteria. This cyanobacterial osmolyte contains substantial carbon and nitrogen, making it a potentially important resource in an environment with fluctuating N and C such as Yawzi Reef (Fig. S1). We did not capture *Trichodesmium* in the 16S rRNA gene amplicon data of this study; however, the presence of this microorganism on these reefs has been visually observed, and the presence of its osmolyte in the exometabolome has been detected in high abundance [75]. We hypothesize that this could be due to viral lysis, grazing, or bloom decay, resulting in an increased release of homoserine betaine. It is key to note that although we observed diel differences in homoserine betaine in each of the sites, not all *Pelagibacter* members had concurrent significant shifts in relative abundance, potentially because of hydrodynamics differently impacting microbiome stability relative to the exometabolome across the sites. Nevertheless, correlation-based approaches, while not definitive, can be used to relate the exometabolome and microbiome and generate within-site and between-ecosystem hypotheses that invite further investigation using more sensitive and directed techniques.

We hypothesized that hydrodynamics strongly influences the microbial communities at Yawzi reef through mixing or flushing. Our hydrodynamic model demonstrated low mid-day presence of coastal bay water at Yawzi reef due to a large influx of offshore water, which likely caused exometabolite and microbial community variability and a more homogenous influx of offshore/oligotrophic microbes at mid-day. This influx of offshore water and more dynamic environment at Yawzi may also explain the decrease in significant exometabolite–microbe correlations compared to Tektite, where a more stable hydrodynamic environment may improve detection of these relationships. The opposing dynamics between exometabolite and microbial dispersion across reef and seagrass could be largely influenced by these hydrodynamic conditions. At the coral reef sites where hydrodynamics were more variable, we see a corresponding decrease in exometabolome similarity but a stable microbial community population. In contrast, at the seagrass site stable hydrodynamic conditions give rise to stable exometabolite profiles, and variable bacterial and archaeal microbial communities. In other studies, metagenomics [76] or metabolomics [77], have been shown to have increased robustness compared to taxonomic composition (ASVs) measurements spatially and across seasonal timescales, due to the redundancy in genes or metabolites across taxonomic lineages. Additional site-level differences that could influence the observed exometabolome and microbial community dispersion patterns include differences in sampling depth, particularly in the shallow sunlight seagrass site (3 m) compared to the deeper coral reefs (8–9 m); environmental differences, such as varying nutrient regimes and inputs; and physical drivers such as tides and residences times.

Our results revealed that site, time of day, and hydrodynamics all strongly influence the microbial communities and exometabolites present within coral reefs and seagrass meadows. While current monitoring strategies rely heavily on visual observations and macro-organismal metrics, our findings highlight the capabilities of ‘omics measurements to detect subtle shifts in the daily rhythms of exometabolite–microbial interactions and that hydrodynamic modeling is useful to further explain the trends. Integrating exometabolites, microorganisms, and hydrodynamics offers a path toward more proactive and informative monitoring frameworks and should be considered in the design of restoration strategies.

## Supplementary Material

Supplementary_material_wrag177

## Data Availability

All MS data are available at MetaboLights under accession number MTBLS9008 (https://www.ebi.ac.uk/metabolights/MTBLS9008), and the sequencing data are available at NCBI SRA under accession PRJNA1380722. A step-by-step BC derivatization protocol is publicly available on protocols.io (dx.doi.org/10.17504/protocols.io.biukkeuw). All MATLAB scripts used for processing the Skyline outputs are available on GitHub (https://github.com/KujawinskiLaboratory/SkyMat).
