# Supplementary material for: Spatiotemporal and hydrodynamic influences on microbial and exometabolite dynamics in coral reef and seagrass ecosystems
Source: ISME J. 2026 Jul 6;20(1):wrag177. doi: 10.1093/ismejo/wrag177 (PMC13403184; doi:10.1093/ismejo/wrag177)
Supplement: Supplementary_material_wrag177 [file supplementary_material_wrag177.zip › Garcia_ISMEJ_accepted_Supplemental.docx]

**Supplementary Information****:** Spatiotemporal and hydrodynamic influences on microbial and exometabolite dynamics in coral reef and seagrass ecosystems

**Running Title:** Drivers of coastal seawater dynamics

**Authors:** Brianna M. Garcia^1^, Sharon L. Grim^1^, Yan Jia^2^, Laura Weber^1^, Cynthia C. Becker^1,4^, Mallory Kastner^1,3^, Gretchen J. Swarr^1^, Melissa C. Kido Soule^1^, Aushaun Brown^5^, Weifeng Zhang^2^, Elizabeth B. Kujawinski^1*^, Amy Apprill^1*^

***Corresponding Author(s):** Amy Apprill ([aapprill@whoi.edu](mailto:aapprill@whoi.edu)) and Elizabeth B. Kujawinski ([ekujawinski@whoi.edu](mailto:ekujawinski@whoi.edu))

**Author emails and ORCIDs:**

Brianna M. Garcia - [brianna.garcia@whoi.edu](mailto:brianna.garcia@whoi.edu); <https://orcid.org/0000-0003-2073-8144>

Sharon L. Grim - [sharon.grim@whoi.edu](mailto:sharon.grim@whoi.edu); <https://orcid.org/0000-0003-4215-7519>

Yan Jia - [yan.jia@whoi.edu](mailto:yan.jia@whoi.edu); https://orcid.org/0000-0002-3306-4110

Laura Weber - [laura.gray@whoi.edu](mailto:laura.gray@whoi.edu); <https://orcid.org/0000-0003-4957-2758>

Cynthia C. Becker - [cbecker3@ithaca.edu](mailto:cbecker3@ithaca.edu); <https://orcid.org/0000-0002-7174-2306>

Mallory Kastner - [mallory.kastner@whoi.edu](mailto:mallory.kastner@whoi.edu); <https://orcid.org/0000-0003-1244-1519>

Gretchen J. Swarr - [gswarr@whoi.edu](mailto:gswarr@whoi.edu); <https://orcid.org/0000-0001-6690-1897>

Melissa C. Kido Soule - [msoule@whoi.edu](mailto:msoule@whoi.edu); <https://orcid.org/0000-0001-6846-7262>

Aushaun Brown - [aushaun.brown@students.uvi.edu](mailto:aushaun.brown@students.uvi.edu)

Weifeng Zhang - [wzhang@whoi.edu](mailto:wzhang@whoi.edu); <https://orcid.org/0000-0002-2819-1780>

Amy Apprill - [aapprill@whoi.edu](mailto:aapprill@whoi.edu); <https://orcid.org/0000-0002-4249-2977>

Elizabeth B. Kujawinski - [ekujawinski@whoi.edu](mailto:ekujawinski@whoi.edu); <https://orcid.org/0000-0001-8261-971X>

**Affiliations:**

^1^Department of Marine Chemistry and Geochemistry, Woods Hole Oceanographic Institution, Woods Hole, Massachusetts, United States;

^2^Department of Applied Ocean Physics & Engineering, Woods Hole Oceanographic Institution, Woods Hole, Massachusetts, United States;

^3^The MIT-WHOI Joint Program in Oceanography/Applied Ocean Science and Engineering, Cambridge and Woods Hole, Massachusetts, United States;

^4^Department of Biology, Ithaca College, Ithaca, New York, United States;

^5^University of the Virgin Islands, 2 John Brewers Bay, St. Thomas, U.S Virgin Islands

**Corresponding Author(s) Mailing Address:**

Amy Apprill, Department of Marine Chemistry and Geochemistry, Woods Hole Oceanographic Institution, 266 Woods Hole Road, Mailstop #4, Woods Hole, MA, 02543, United States

Elizabeth B. Kujawinski, Department of Marine Chemistry and Geochemistry, Woods Hole Oceanographic Institution, 266 Woods Hole Road, Mailstop #4, Woods Hole, MA, 02543, United States

**Supplemental Methods:**

Materials:

Hydrochloric acid (HCl), acetone, methanol (MeOH), acetonitrile (MeCN), benzoyl chloride (BC; 99%, ACROS Organics), and sodium hydroxide (NaOH) were purchased from Fisher Scientific (Optima). Phosphoric acid (85%, ACS reagent grade), ^13^C_6_-ring-BC (99% atom ^13^C), and internal standards were purchased from Sigma-Aldrich. Stable isotopically labeled - internal standards matched to each targeted compound (SIL-IS) were prepared with ^13^C_6_-ring-BC. Deionized water was obtained from a Milli-Q system (Millipore; resistivity 18.2 MΩ at 24 °C, TOC < 1 μM). Samples and reagents were stored in acid-washed and combusted (at least 4 h at 450°C) glassware. BC and all solvents were transferred using combusted glass Pasteur pipettes. Primary stocks and mixes were stored at −20°C. The working reagent (5% BC in acetone) was prepared fresh daily.

Targeted metabolomics UHPLC-MS/MS data collection:

Reverse phase (RP) chromatography was performed according to previously published protocols [1]. Briefly, using an ultra-high performance liquid chromatography system (UHPLC; Vanquish, Thermo Scientific, Waltham, MA, USA), separation was achieved on a Waters Acquity HSS T3 column (100Å, 1.8 µm particle size, 2.1 mm X 100 mm) equipped with an Acquity HSS T3 VanGuard pre-column (100Å, 1.8 µm particle size, 2.1 mm X 5 mm) coupled to a heated electrospray ionization source (H-ESI) and an Orbitrap mass spectrometer (Orbitrap Fusion Lumos, Thermo Scientific). Chromatography gradient, mass spectrometer settings, and quality assurance and control (QA/QC) are described in detail elsewhere [2].

Quality Assurance and Quality Control (QA/QC):

Thorough QA/QC was conducted as described in prior publications [2] to ensure the reliability of the metabolites reported herein, with 45 metabolites retained post-QA/QC. Sample outliers were determined through a combination of calculations which included (1) the Euclidean distance between each sample within a site at a given sampling time, (2) the 95% confidence interval (CI) for each site/sampling time non-metric multidimensional scaling (NMDS) plot, and (3) Bray-Curtis dissimilarity distance from the site average within a given sampling time. Sample outliers were removed conservatively, as biological variation across replicates was anticipated. Sample #73 (biological replicate of the seagrass sampled on day three at mid-day) was removed as an outlier as it deviated outside the 1.5 times the interquartile range +/- the upper (75th) quartile and fell outside the 95% confidence interval of the seagrass mid-day sampling group and clustered on top of the coral reef sample groups.

16S rRNA gene sequencing data processing:

Amplicon libraries were analyzed using the DADA2 (v. 1.26.0) package in R. Read pairs were assessed for quality, trimmed of primers, and used to estimate sequencing error rates (filterAndTrim: truncLen = c(210, 160), maxN = 0, matchIDs = TRUE, multithread = 12, maxEE = c(2,2), truncQ = 2, rm.phix = TRUE, compress = TRUE). Read pairs were merged to cover the length of the 16S rRNA gene V4 region (mergePairs: minOverlap = 12), and the resulting amplicon sequence variants (ASVs) were checked for chimeras (removeBimeraDenovo: method = "consensus", multithread = nthreads). Taxonomy was assigned to ASVs using the Silva v.138.1 database [3] (assignTaxonomy: minBoot = 50, tryRC = TRUE, multithread = 12). R package decontam (v. 1.18.0) was used to identify putative contaminant sequences using negative control samples from DNA extraction and PCR amplifications (isContaminant: method = "minimum", threshold = 0.1). We examined the distribution of putative contaminants at both the species level and ASV level, to account for endemism and biological variability between sites. This method identified 339 potential contaminant ASVs representing 104 different species. After removing sequences assigned to potential contaminants (n = 7,617) and of eukaryotic origin (chloroplasts, mitochondria, and eukaryotes: n = 171,607), the dataset of 72 samples retained 6,136,788 sequences representing 10,274 ASVs. Due to the nuances of taxonomic databases, for several ASVs taxonomic assignment was less specific beyond the class level. In order to include such ASVs that were also abundant in the study, the last known taxonomic lineage was propagated to the genus level. For example, of the 498 ASVs assigned to the Pseudomonadales family (Gammaproteobacteria) 269 had taxonomic assignments to the genus level. This excluded 49 abundant ASVs assigned at the family level to the SAR86 clade. Thus, to examine taxonomic diversity at ecologically relevant scales, the family-level SAR86 clade was also included.

Statistical Analysis:

*Metabolomics statistical analyses*

Statistical analyses were carried out in R Studio (2024.09.1+394) running R (4.3.2). Bray Curtis dissimilarity between metabolomic samples was calculated and visualized by non-metric multidimensional scaling (NMDS) using the 'vegan' [4] and 'ggplot2' [5] packages. To accommodate the wide range of inter-site diversity, we compared within-site NMDS visualizations from a distance matrix using all samples, as well as site-specific distance matrices. A Permutational Multivariate Analysis of Variance (PERMANOVA) using Bray Curtis distance matrices was carried out on log2(x+1) exometabolite concentrations and environmental categorical variables using the adonis2 function in the ‘vegan’ package. A distance-based redundancy analysis (dbRDA) was used to test categorical and continuous environmental and biological factors against the metabolomics data using Bray-Curtis distances calculated by vegan::vegdist and the vegan::capscale functions. A Kruskal-Wallis test (stats::kruskal.test) was used to test for significant exometabolites between dawn and mid-day samples within sampling sites. A Kruskal-Wallis test (stats::kruskal.test) followed by Pairwise Wilcoxon Rank Sum tests (stats::pairwise.wilcox.test) were conducted to determine significant exometabolites across sites within a sampling time. *P* values were adjusted to account for multiple comparisons using the Benjamini-Hochberg correction using an implementation of stats::p.adjust(method = “fdr”), and exometabolites were considered significant with a false discovery rate (FDR) adjusted *P* value < 0.05.

*Microbial statistical analyses*

Amplicon sequence data were analyzed in RStudio (v 2024.09.0) running R (v4.2.2) with the following packages: tidyverse (v 2.0.0), phyloseq (v 1.42.0), corncob, DESeq2, vegan (v 2.6.1), ashr (v. 2.2.63), boot (v. 1.3.31), spiec.easi (v 1.1.3), and NetCoMi (v 1.1.0). Alpha-diversity estimates (vegan::rarecurve) (vegan v 2.6.1) for samples within each site used per-sample raw sequence counts rarefied to the lowest sequence depth observed within each site. To identify the most consistent and statistically robust data transformation for amplicon sequence variant (ASV) data, we compared results from combinations of three different data transformations and four dissimilarity metrics applied to ASV profiles, used for community beta diversity via nonmetric multidimensional scaling (NMDS), permutation of analysis of variance (PERMANOVA) via adonis2, and Spearman correlation analysis with the exometabolome (Table S13). Unmanipulated raw sequence counts from ASV profiles as well as relative abundances (i.e., contributions of observed ASVs were normalized to 100% in each sample) were input to data transformations: centered log-ratio (“CLR”) with a pseudocount of 1 (raw counts) or 0.001% (relative abundance) [6], or robust centered log ratio (“rCLR” or “robust CLR”) [7]. The relative abundances without further transformation were also examined. Dissimilarity metrics implemented in vegan::vegdist and vegan::adonis2 were: Bray-Curtis, Morisita-Horn, Euclidean, and Chord (Table S13). NMDS and PERMANOVA via adonis2, implementations described below, were used to select the two best transformations for microbiome-specific results, ASV relative abundances and rCLR transformed counts. They were further compared via Spearman correlations with exometabolite profiles, as described below, to identify the most appropriate data transformation for use in all statistical analyses.

After statistical cross-comparison described above determined that a relative abundance-based approach was most suitable for our analyses (Supplemental Results), Morisita-Horn dissimilarity was calculated between samples’ relative abundances of the amplicon sequence variants (ASVs) and used for community beta diversity assessments. As with the exometabolomes, NMDS (vegan::metaMDS) visualizations were constructed from a distance matrix using all samples, as well as site-specific distance matrices to accommodate the wide range of inter-site diversity. R packages corncob (v. 0.4.1) and DESeq2 (v. 1.38.3) were used to identify significantly differentially abundant ASVs between pairs of sites at the same sampling time, and between sampling times within the same site (total of 9 contrasts). Each package handles data and statistical testing differently; corncob uses one of three tests (Wald, log-likelihood ratio, or Rao) to examine differential abundance and variability in abundance of ASVs between groups of samples. By default, corncob filters for significant taxa using an implementation of stats::p.adjust(method = “fdr”), which assumes the observations are independent. A more conservative approach was used to filter the results, by modeling the local false discovery rates (LFDR) (ashr::qval.from.lfdr) from the computed *P* values, and calculating the 1%, 5%, and 10% LFDR quantiles. After examining the *P* values distributed in those quantiles, the corresponding adjusted *P* values, modeled estimates and coefficients for all observed taxa, a 5% (α = 0.05) experiment-wise error rate was determined to be most effective at culling putative false positives that arose from multiple tests of non-independent observations. This post-test filtering step retained 708 observations of 207 unique ASVs, with a maximum corresponding *P* value of < 0.0539). DESeq2 normalizes ASV relative abundance and sample abundance by sampling depth and number of samples, sets the per-ASV expected abundance (null hypothesis) as the mean abundance across the input samples, and can implement a variety of metrics for post-test corrections beyond constraining the false discovery rate. Similar to post-test handling of corncob results, raw *P* values from DESeq2::results(alpha = 0.05) were used to model 1%, 5%, and 10% LFDR quantiles. An experiment-wise false discovery rate of 10% (α = 0.10) was used to filter the corresponding adjusted *P* values and shrunken log2(fold change) estimates from DESeq2::lfcShrink(type = "ashr"), yielding 1306 observations of 370 unique ASVs with a maximum corresponding *P* value < 0.0497. Results from both methods were compared; 189 distinct ASVs unique to DESeq2 and 26 distinct ASVs unique to corncob were identified as significantly differentially abundant with respect to method. Across both methods, 181 unique ASVs were flagged to be significantly differentially abundant in paired sample groups, which we retained for further analyses.

*Concurrent exometabolome and microbiome diversity analyses:*

For exometabolomes and microbiomes, a permutation of analysis of variance (PERMANOVA) via vegan::adonis2 was calculated to assess the impact of site, sampling time, and sampling day on exometabolite and microbial population diversity. As with the NMDS, the input pairwise dissimilarity matrices to PERMANOVA were constructed from Bray-Curtis dissimilarity on log2(x+1) transformed exometabolite profiles, and Morisita-Horn dissimilarity on ASV relative abundances. Estimates of variation were calculated from individual terms (site, time, and day), as well as interactions of terms. Interaction terms “sampling time x site” and “sampling time x sampling day x site” yielded negative estimates in the original model. As recommended, they were pooled one at a time with the term with the lowest estimate of variation, “sampling time x sampling day”, and the model was re-run until no negative estimates were generated [8]. The final model included the interaction terms “sampling day x site” and a pooled term (“sampling time x sampling day”, “sampling time x site”, and “sampling time x sampling day x site”), in addition to the original source variables. To estimate the 5% FDR threshold for significance, a custom permutation model using the boot::boot function generated a modeled distribution of *P* values calculated from 9999 permutations of randomly subsampled data.

The impact of site, sampling time, and sampling day on microbial and exometabolite community diversity was assessed using bootstrapped t-tests (via stats::t.test) and Kruskal-Wallis rank sum tests (via stats::kruskal.wallis). For each comparison, the mean difference in beta diversity, here reported as Δ$\beta$, was calculated from groups of sample beta diversity values. The distribution of beta diversities was assessed using vegan::betadisper(type = “median”, sqrt.dist = FALSE), which calculates the dispersion or distance between each sample’s coordinates in NMDS and a group representative centroid, in this study using the spatial median. The median of beta dispersion values for each group is referred to here as the median $\sigma_{\beta}^{2}$. Using beta diversity values quantifies similarity between groups of samples, whereas beta dispersion comparisons convey if the degree of variability within a specific group of samples is statistically different from another group.

*Exometabolite-microbe correlations:*

Site-specific spearman correlations were conducted between log2(x+1) transformed exometabolite concentrations and ASV relative abundances. Because some microbial samples had technical replicates in the exometabolite samples (one filter corresponding to multiple filtrate samples), technical replicate exometabolome samples were averaged to ensure a one-to-one relationship between the exometabolome and microbiome data. Due to the increased granularity of subsetting the site-specific datasets for correlation analyses, the impact of data granularity was assessed on adjusted *P* values (stats::p.adjust) and FDR q-values (ashr::qval.from.lfdr) (Table S12), demonstrating that with increased granularity of the site-specific datasets that BH-adjusted *P* values < 0.05 corresponded to a maximum raw *P* value < 0.0003. For the explorative nature of this analysis, this level of stringency was deemed unnecessary. Thus, a Spearman’s rho (ρ) of |0.5| and the *P* value corresponding to a 1% FDR were selected as the criteria for significant correlations.

Hydrodynamic modeling:

The St. John hydrodynamic model is based on the widely used Regional Ocean Modeling System (ROMS) and has been used to simulate fine-scale circulation in the St. John area, including the numerous bays, with a horizontal grid resolution of 50 m [9]. The model resolves the fine-scale coastal bathymetry, including the shallow shelf, around St. Thomas, St. John, and the British Virgin Islands. To simulate the time-evolving, three-dimensional flows around the islands and in the bays, the model captures vertical water mixing as well as bottom friction. The model is forced on the lateral open boundaries by calibrated realistic fields of tides, temperature, salinity, and velocity from large-scale ocean models and at the surface by hourly meteorological conditions from a global atmospheric model. Results of the model hindcast simulation in 2016-2022 were validated against historical observations on the St. John coastal region.

**Supplemental Results and Discussion:**

*Alternative data transformations and dissimilarity distances*

When using CLR-transformed ASV counts, regardless of dissimilarity metric, NMDS models either never converged after 100 iterations and/or had poor stress (> 0.1), due to the sparseness inherent in microbiome sequencing data [6]. Both Bray-Curtis and Morisita-Horn dissimilarity metrics calculated from ASV relative abundance (hereafter referred to as “BC” and “MH”, respectively) yielded NMDS models with stress = 0.075 (Table S13). Procrustes analysis (vegan::protest), which tests the similarity between two NMDS solutions such as models obtained from two different data transformations/dissimilarity metrics, showed significant and high correlation between BC and MH distance matrices (t_0_ = 0.94, p < 0.001; Table S13). Robust CLR transformation of ASV sequence counts followed by Euclidean distance, functionally equivalent to robust Aitchison distance [7], had the lowest model stress of the options (0.055). The Robust Aitchison model was significantly and strongly correlated to BC (Procrustes, t_0_ = 0.74, p < 0.001; Table S13) and MH (t_0_ = 0.65, p < 0.001; Table S13) models. Both MH and BC suffer biases when sampling fraction is uneven, meaning that the presence of nonzero abundance of ASVs per sample strongly varies, but BC performs slightly worse than MH when data are sparse [10]. Across samples, sequence counts ranged ~10-fold (min: 13,915, max: 114,423), and nonzero observations of ASVs ranged from 169 to 827 ASVs. For our data, Morisita-Horn had fewer biases than BC and similar beta diversity results to robust Aitchison, thus these were the two combinations of data transformation and dissimilarity distance for adonis2 PERMANOVA: relative abundances with Morisita-Horn dissimilarity distance, and robust Aitchison (robust CLR and Euclidean dissimilarity distance).

Permutation of analysis of variance (PERMANOVA) via vegan::adonis2 yielded similar results between the two examined data transformations (Table S13). Between 44.0% and 47.3% of variation in the adonis2 model was explained by inherent differences between the three sites. The MH model suggested 21.2% of variability was due to sampling time, and slightly less (14.7%) attributed to sampling day. In contrast, the robust Aitchison model flipped these percentage contributions with sampling day attributed to more (21.4%). When considering interactions of terms, both models agreed that site-specific sampling day explained approximately 8.0% of variability in community beta diversity, and other combinations of interacting variables contributed up to 3.1% (MH) and 5.5% (rCLR). Finally, the adonis2 model based on MH had a lower residual (5.3%) compared to rCLR (6.2%), indicating that the examined environmental variables accounted for a higher proportion of variability in the adonis2 MH model. Cumulatively, Morisita-Horn dissimilarities based on relative abundances performed slightly better than Euclidean distances from robust centered log-ratio transformed microbiome profiles.

*Robust centered log-ratio skews natural microbiome data in correlative analyses*

Within each sampling site, Spearman correlations (stats::cor.test) were calculated between paired profiles of log2(x+1) metabolite concentrations, and microbiome profiles that were either rCLR-transformed or normalized via relative abundance (Table S13). We focused on only strong correlations (|r| ≥ 0.5) and implemented multiple test corrections on the significant *P* values: Benjamini-Hochberg (BH) adjustment implemented in stats::p.adjust; and calculating quantiles of *P* values using ashr::qval.from.lfdr to determine a 1% false discovery rate (FDR). Using ASV relative abundances, only 38 strong Spearman correlations had adjusted *P* value < 0.05, and those were observed in only the seagrass samples. The alternative 1% FDR curation of *P* values recovered 442 strong correlations in Tektite reef and 373 correlations in Yawzi reef, as well as an additional 942 correlations in Lameshur seagrass (Table S13). In comparison, BH-adjustment of *P* values from rCLR-transformed microbiome profiles identified thousands of strong and significant correlations in each site. In Lameshur seagrass, 12,802 strong correlations had adjusted P values < 0.05, followed by 11,811 correlations in Yawzi reef and 4,125 correlations in Tektite reef. As we did with the relative abundance-based data, applying the same 1% FDR approach to these *P* values removed all correlations from Lameshur seagrass, and reduced the number of correlations in the reef sites to 893 in Tektite and 1,066 in Yawzi.

While it was necessary to use 1% FDR to identify significant Spearman correlations using relative abundances, Benjamini-Hochberg *P* value adjustment was more appropriate for the rCLR-based approach. The discrepant recovery of correlations between the two microbiome transformations is due to *P* value distributions between the two correlations sets. From the relative abundance model, in all three sites the *P* value cutoff for a 1% FDR was *P <* 0.02 (Table S13). In that set of *P* values, the corresponding *minimum* BH-adjusted *P* value was between 0.05 (Lameshur) and 0.09 (Yawzi). With rCLR-transformed data, the *P* value cutoff for 1% FDR was between *P* = 0.002 (Yawzi) and 0.01 (Tektite). The *maximum* BH-adjusted *P* value corresponding to 1% FDR was between 0.03 (Yawzi) and 0.10 (Tektite). Since a 1% FDR for Lameshur seagrass correlations discarded all correlations, we calculated the 1.2% FDR *P* value = 0.00000005 retained only 40 correlations and corresponded to a *maximum* BH-adjusted *P* = 0.00001. Using 1% FDR to reduce the probability of false positives was overly strict on rCLR-based correlations, whereas BH adjustment was too conservative with relative abundance-based results. Since consensus on a multiple test correction method was not possible between the two datasets, we used the best individual approach to filter for comparable numbers of correlations for further examination.

After multiple test corrections as described above, we identified |r| thresholds to filter for the strongest, significant correlations. In the relative abundance-based set of results, the median |r| was 0.59 (Lameshur), 0.58 (Tektite), and 0.55 (Yawzi); the maximum |r| was 0.94, 0.99, and 0.98, respectively. The median and maximum |r| being within ± 0.025 across all three sites highlights similar distributions of |r|. Within each site, |r| = 0.8 represented the 85^th^- to 95^th^- percentile of observed correlations (e.g. the top 5-15%), so we used this threshold to focus on the strongest correlations |r| ≥ 0.8 (Lameshur: 28, Tektite: 23; Yawzi: 49). In contrast, the median |r| based on rCLR transformed data was 0.71 (Lameshur), 0.68 (Tektite), and 0.65 (Yawzi), but the maximum |r| was 0.91, 0.78, and 0.76 respectively. At |r| ≥ 0.8 only 1,019 correlations from rCLR-based data remained, and all those were observed in Lameshur Seagrass. Therefore, we examined the top 5% of correlations in the rCLR dataset, corresponding to customized thresholds for each site: |r| ≥ 0.81 in Lameshur Seagrass yielding 648 correlations, |r| ≥ 0.71 in Tektite Reef retaining 220 correlations, and |r| ≥ 0.73 in Yawzi Reef keeping 763 correlations.

In the dataset of Spearman correlations obtained from ASV relative abundances, the 100 significant and strong correlations were generated between 14 metabolites and 88 unique ASVs representing 54 distinct taxonomic lineages. This included two ASVs with archaeal lineages (Thermoplasmata; Marine Group II) that were observed in Yawzi Reef to be correlated with exometabolites homoserine betaine, 5'AMP, and 3’AMP. In comparison, the top 5% of rCLR correlations included 1,414 unique ASVs from 409 distinct taxonomic lineages, correlating to only 5 exometabolites: pantothenic acid, homoserine betaine, putrescine, sarcosine, and arginine. The 1,414 ASVs resolved into 743 distinct taxonomic lineages and included 219 archaeal ASVs with 10 taxonomic lineages. Nearly half of the top 5% of observed correlations with Archaea were between Nanoarchaeia; Woesearchaeales ASVs and sarcosine (112 of 219). Nanoarchaeia ASVs identified as significantly correlated with metabolites within the rCLR data were each less than 0.015%, and frequently < 0.001%, of microbial sequences in any sample. More broadly, between 157 and all the 219 archaeal ASVs that were considered significantly correlated via rCLR transformed data, had 0 amplicon counts in any sample.

In addition to the inclusion of rare or sparse ASVs when using rCLR data, we observed a disproportionately small group of significantly associated metabolites. Even at a lower stringency of |r| filtering, such as in the top 15% of rCLR correlations, 3x as many correlations were retained but did not expand beyond the same 5 metabolites being strongly and significantly correlated with microbes. While such a small group of metabolites may be used to support cross-network associations, for example, this may also be an artefact of statistical handling. Microbiome data, especially relative abundance profiles, are not normally distributed and overwhelmingly tend to sparseness. Robust centered log-ratio can make these data more suitable for normality-sensitive tests such as PCA and other matrix-based approaches (Martino et al., 2019), by minimizing the effect of zeros (e.g. absence of an ASV) in the data while at the same time putting more “weight” on observations of nonzero ASVs. When rCLR-transformed data are the input to Spearman correlations, a nonparametric test to understand associations between two variables, we observed that the intentional smoothing of zeros/absent ASVs leads to stochastic shifts in their transformed distribution profiles. For example, our dataset included 34 ASVs belonging to *Pelagibacter* (formerly Clade Ia of the SAR11) and over 1,600 ASVs assigned to Woesearchaeales. In each sample, on average 9 *Pelagibacter* ASVs and 1,332 Woesearchaeales ASVs were not observed (i.e. had 0 amplicon counts). Among all nonzero occurrences of *Pelagibacter,* for each ASV the median relative abundance was 9.7% and the median rCLR-transformed value was 0.014 across all samples. In contrast, when any of the Woesearchaeales ASVs were observed in a sample, they had a median relative abundance of 0.10%, but these ASVs had a median rCLR-transformed value of 0.001. When comparing 45 metabolite concentrations to the relative abundances of 169 to 827 nonzero ASVs, each sample has approximately 7.6 x 10^3^ to 3.7 x 10^4^ Spearman correlations to filter through. But with a full suite of ~10,000 ASVs represented in its rCLR-transformed profile, 4.6 x 10^5^ ASV-metabolite pairwise correlations per sample substantially increases the noise when filtering for significant relationships, at the expense of metabolites. This method intended to highlight potentially ecologically relevant associations between microbes and metabolites, does not benefit from rCLR transformation which instead artificially inflates the importance of rare or overwhelmingly sparse ASVs.

Sampling Day Significance

The PERMANOVA models suggested that a minor, but significant contribution to variability in the exometabolome (11.7%) and microbiome (8.3%) was site-specific sampling day (p < 0.05). More broadly, sampling day was found to significantly contribute to the microbiome (14.7% of variability, *P* value < 0.001), yet not the exometabolome (*P* value = 0.246). Sampling day was insignificant in the metabolomics dataset and had the lowest percent contribution (14.7%) of the investigated variables to the microbiome data, thus allowing for samples to be grouped across days based on site and/or sampling time allowing for robust statistics.

Environmental factors

A redundancy analysis (RDA) investigated the influence of various environmental and biological variables on the metabolome composition. Variables tested included total organic carbon (TOC), total nitrogen (TN), and abundances of *Prochlorococcus*, *Synechococcus*, picoeukaryotes, and heterotrophic cells. All measurements were log2(x+1) transformed and tested for colinearity before running the RDA. A strong positive correlation was found between *Prochlorococcus* and *Synechococcus* (0.87), therefore *Synechococcus* measurements were excluded from the RDA to limit variable redundancy. The significance of the individual terms in the RDA model was evaluated using an ANOVA and found no significant variables (*P* value < 0.05) excluding site and sampling time, consistent with previous findings in the PERMANOVA.

**Supplemental Figures:**

**
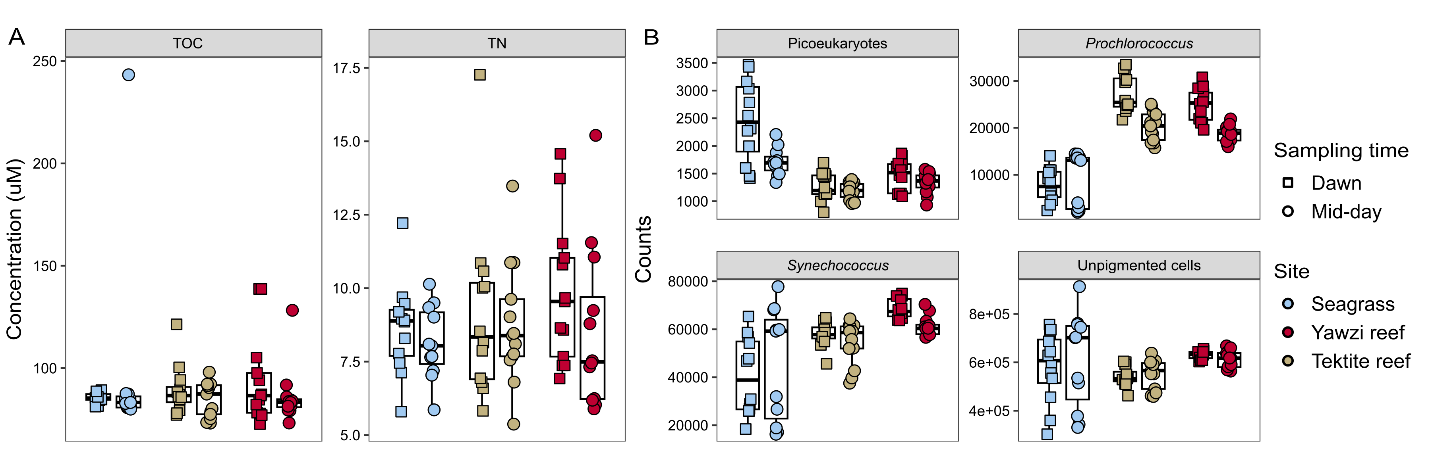
**

**Supplemental Figure 1. Cell counts and bulk dissolved organic carbon and nitrogen measurements.** Boxplots for each sampling site at dawn (squares) and mid-day (circles) over the four consecutive sampling days for (A) bulk water chemistry concentrations in micromolar (uM) of total organic carbon (TOC) and total nitrogen (TN), and (B) flow cytometry counts of picoeukaryotes, *Prochlorococcus*, *Synechococcus*, and unpigmented cells (heterotrophic bacteria and archaea).

**
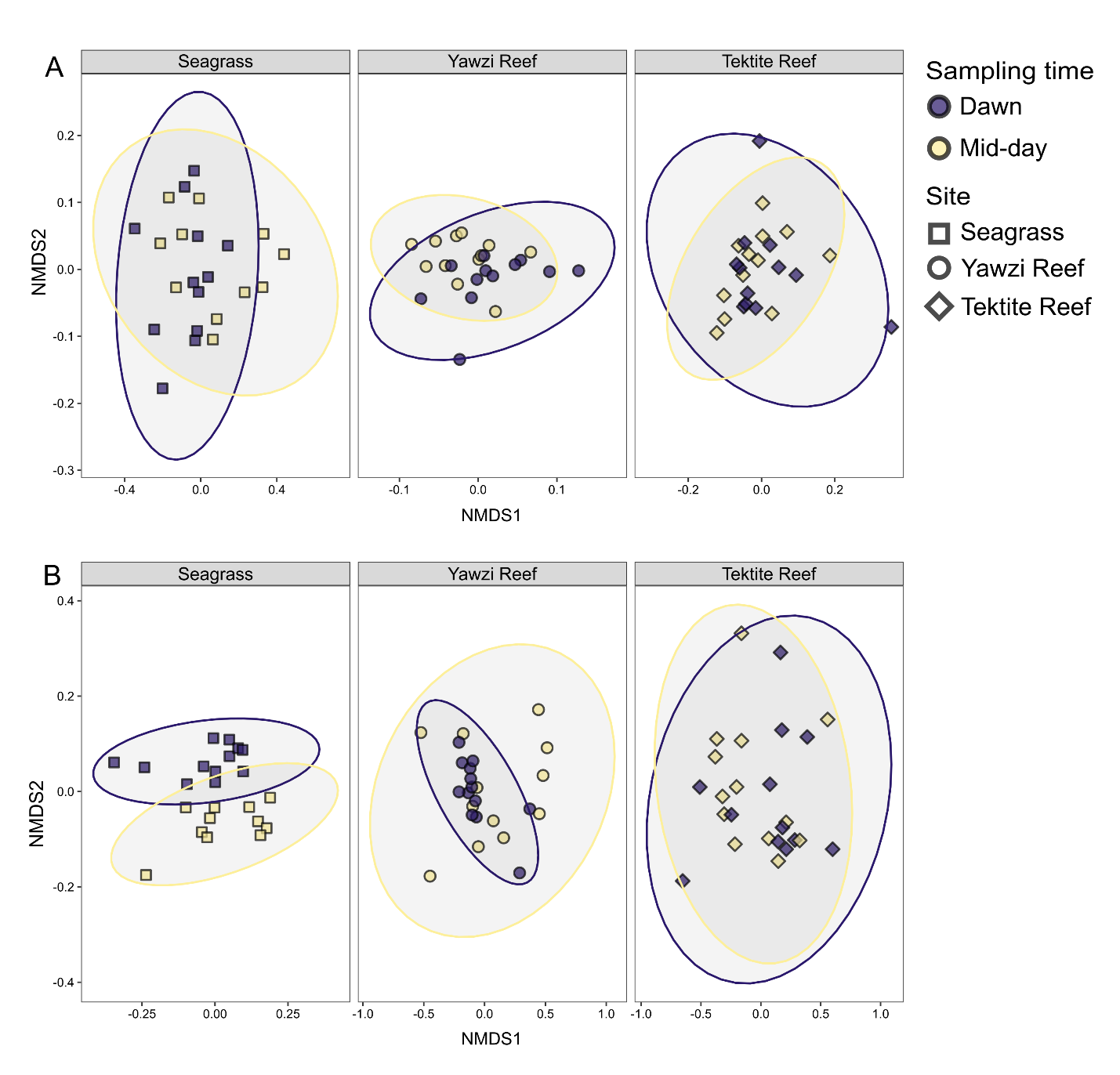
**

**Supplemental Figure 2. Site-specific NMDS ordination plots** of the (A) microbiome and (B) exometabolome are shown using Morisita-Horn and Bray-Curtis dissimilarities, respectively. Confidence intervals (95%) are shown as ellipses for dawn (blue) and mid-day (yellow) sampling times. Sites are separated into their own individual facets and data points are represented by unique shapes per site.

**
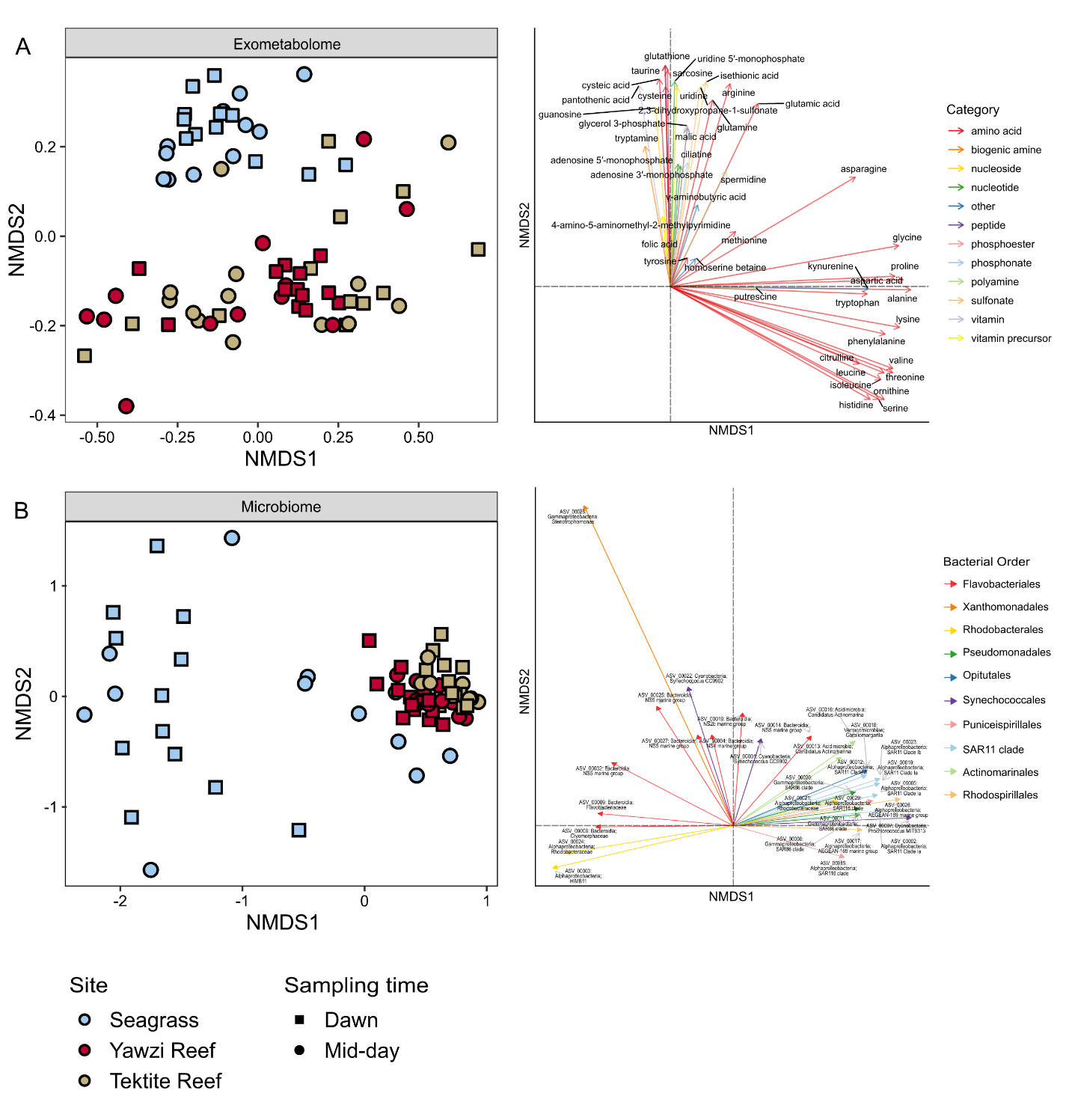
**

**Supplemental Figure 3. Exometabolite and microbial ASV drivers of multi-dimensional separation.** NMDS of microbiomes from seagrass (blue), Yawzi reef (red), and Tektite reef (yellow) as seen in main body Figure 3 with additional side-by-side vector profiles of A) exometabolites and B) microbial ASVs. Exometabolite vectors are color-coded by metabolite compound class with all 45 quantified metabolites displayed. ASV vectors were filtered to only highlight the ASVs that were significantly differentially abundant (SDA). Of the 181 SDA ASVs, 36 ASVs were considered the most abundant and frequently observed and displayed in panel B. ASV vectors are color-coded by bacterial order.

**
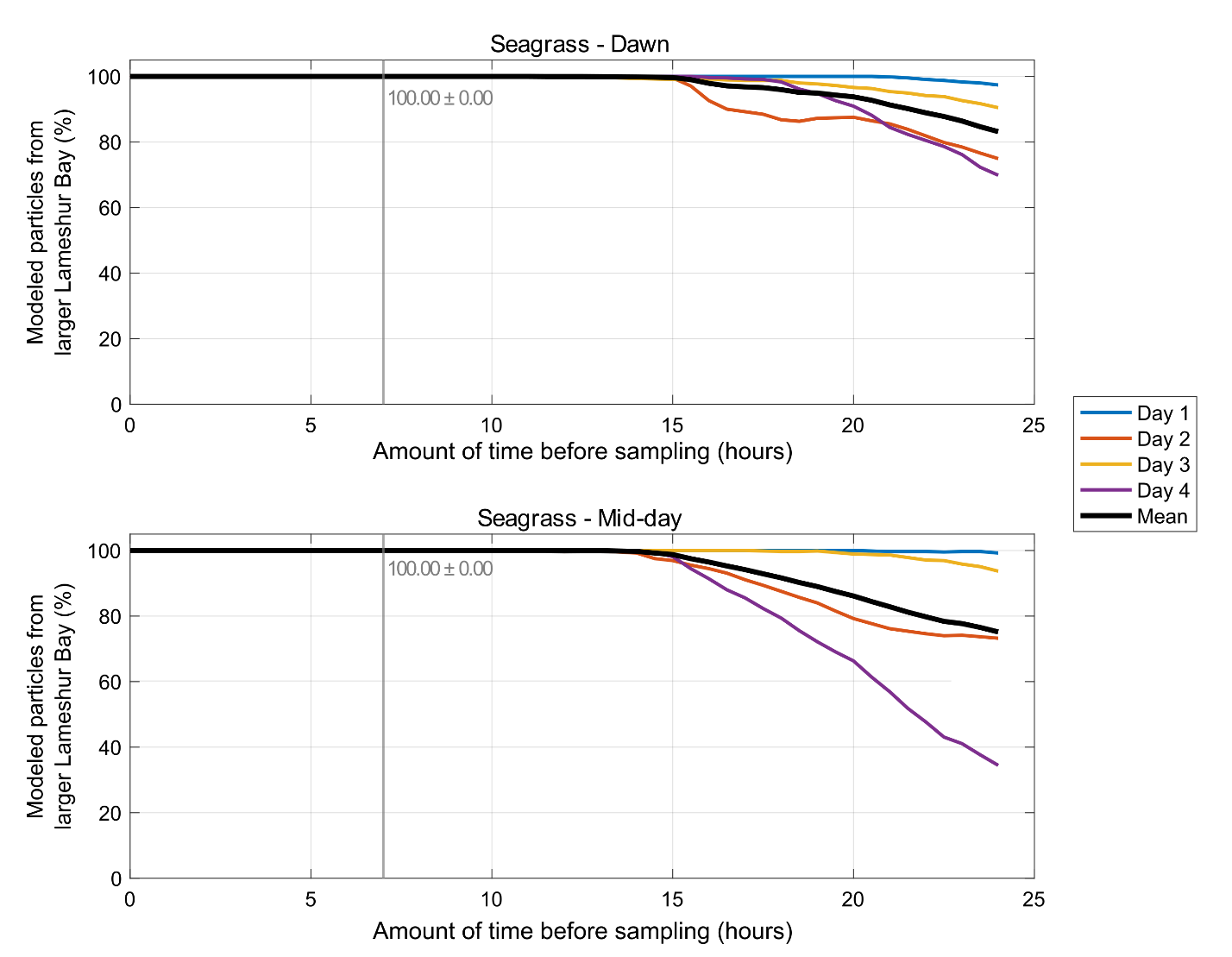
**

**Supplemental Figure 4.** **Sources of water samples in the Seagrass.** Hydrodynamic and particle-tracking models were used to identify the origin of the water samples. Here we show the percentage of modeled particles from the larger coastal region of Lameshur Bay (y-axis) looking backwards in time where the number of hours prior to sampling is depicted on the x-axis. Particle-tracking simulations were carried out for each of the four sampling days, with the average shown in solid black. The average percentage of modeled particles from the larger Lameshur Bay region at seven hours prior to sampling (the approximate number of hours between our sampling times) is shown as a vertical grey line with the average and standard deviation values of that timepoint shown on the plot. Separate simulations were constructed for the dawn and afternoon sampling periods.

**
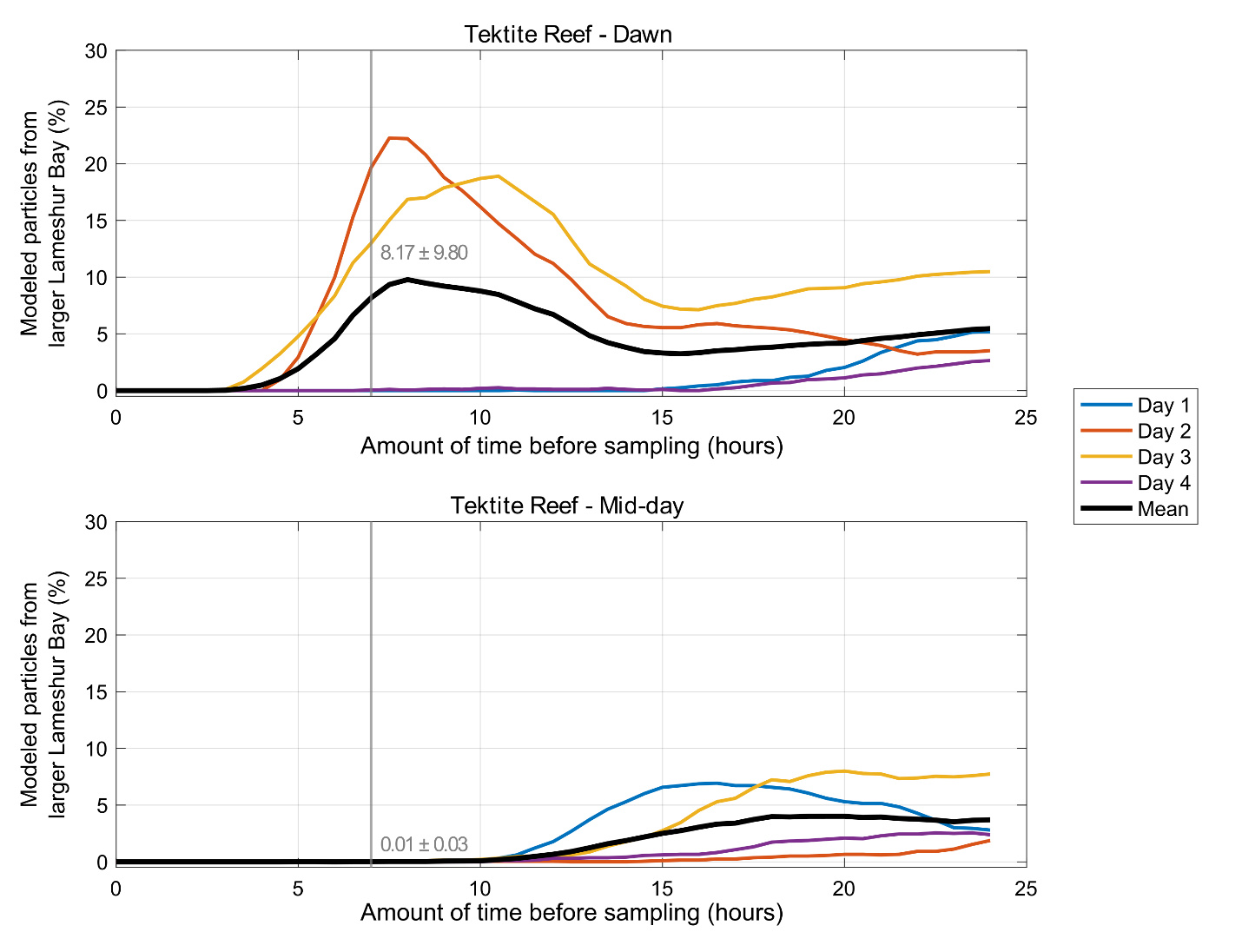
**

**Supplemental Figure 5. Source of water samples at Tektite Reef.** Hydrodynamic and particle-tracking models were used to identify the origin of the water samples. Here we show the percentage of modeled particles from the larger coastal region of Lameshur Bay (y-axis) looking backwards in time where the number of hours prior to sampling is depicted on the x-axis. Particle-tracking simulations were carried out for each of the four sampling days, with the average shown in solid black. The average percentage of modeled particles from the larger Lameshur Bay region at seven hours prior to sampling (the approximate number of hours between our sampling times) is shown as a vertical grey line with the average and standard deviation values of that timepoint shown on the plot. Separate simulations were constructed for the dawn and afternoon sampling periods.

**
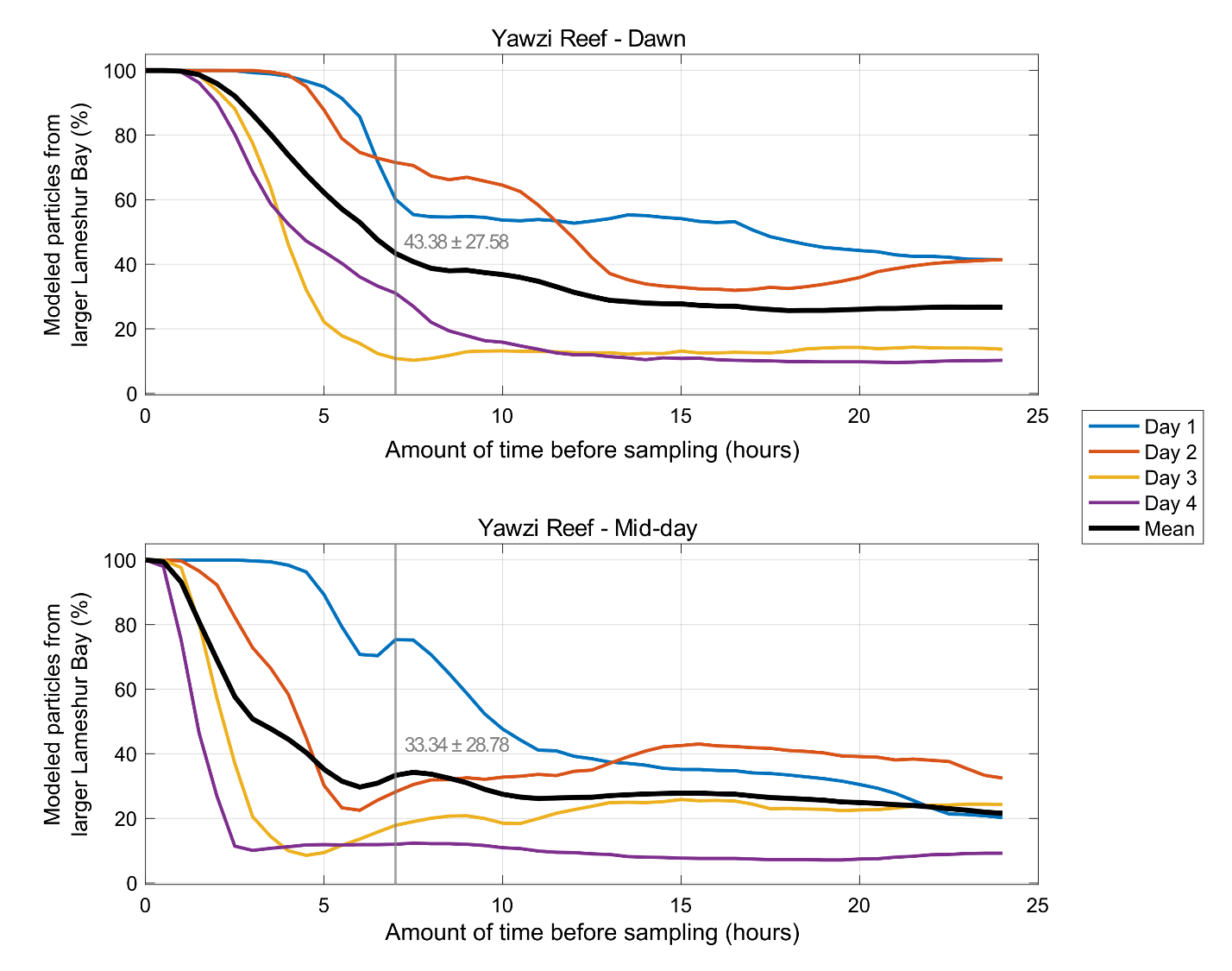
**

**Supplemental Figure 6. Source of water samples at Yawzi Reef.** Hydrodynamic and particle-tracking models were used to identify the origin of the water samples. Here we show the percentage of modeled particles from the larger coastal region of Lameshur Bay (y-axis) looking backwards in time where the number of hours prior to sampling is depicted on the x-axis. Particle-tracking simulations were carried out for each of the four sampling days, with the average shown in solid black. The average percentage of modeled particles from the larger Lameshur Bay region at seven hours prior to sampling (the approximate number of hours between our sampling times) is shown as a vertical grey line with the average and standard deviation values of that timepoint shown on the plot. Separate simulations were constructed for the dawn and afternoon sampling periods.

**
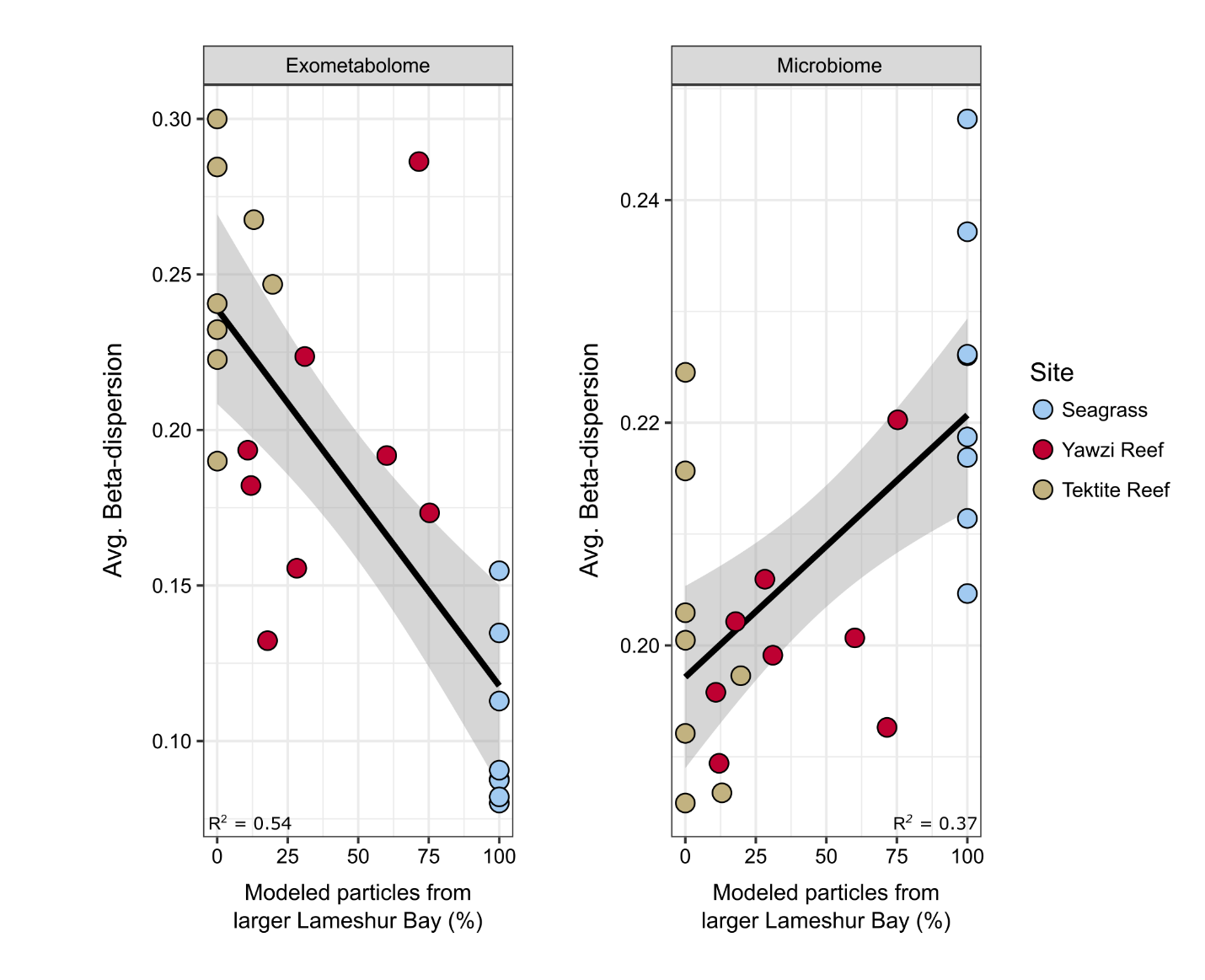
**

**Supplemental Figure 7. Beta-dispersion is related to source water percentage.** Linear model of the average percent of modeled particles from the larger Lameshur Bay region 7-hours prior to water sampling based on the hydrodynamic and particle-tracking models compared against the calculated average beta-dispersion at each sampling site for the exometabolome and microbiome.

**
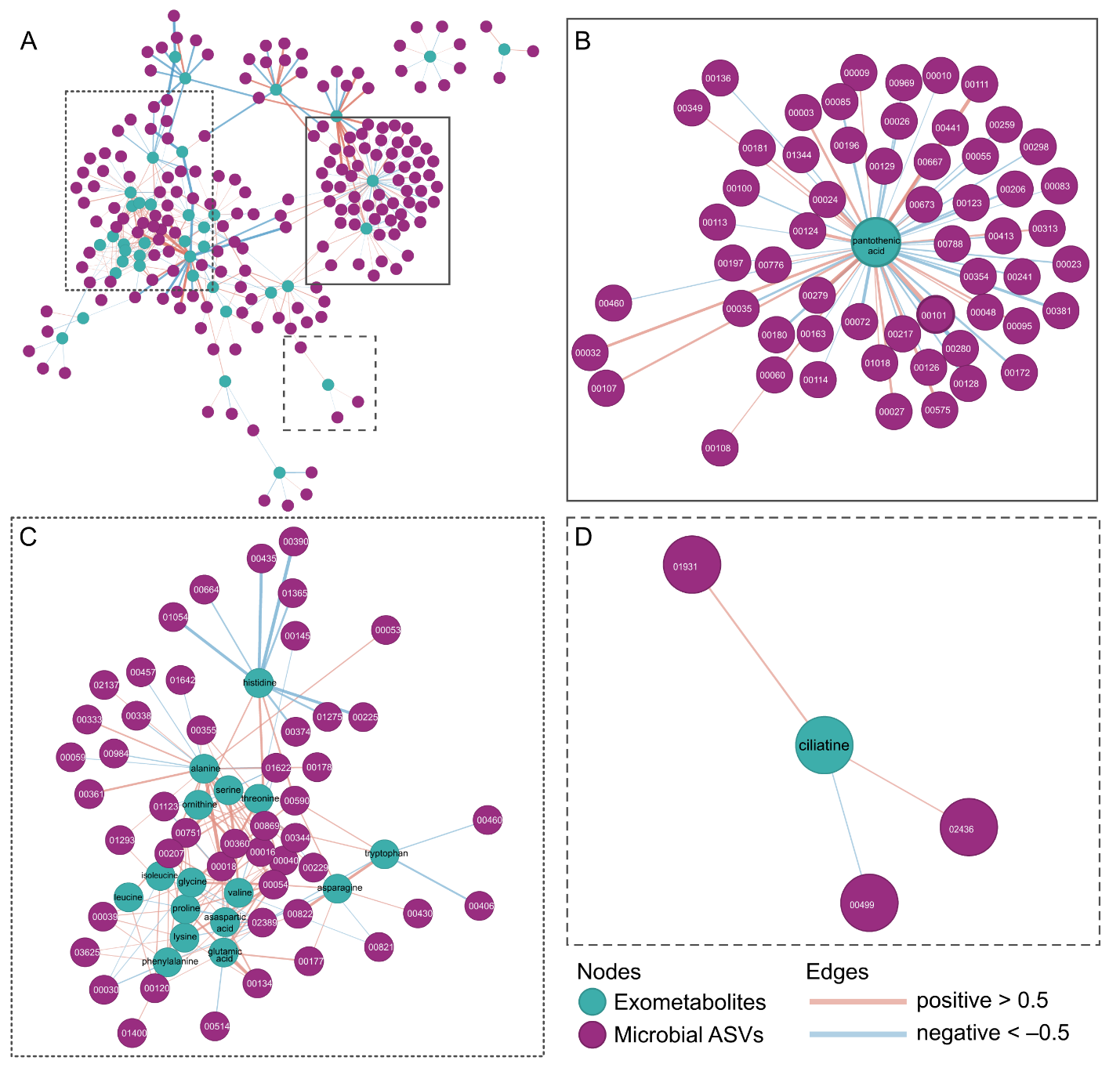
**

**Supplemental Figure 8. Correlations between exometabolites and microbial taxa enable hypothesis generation about ecosystem dynamics.** A) Complete correlation network showing all significant exometabolite-ASV correlations at Yawzi reef using Spearman correlations between exometabolite concentrations and ASV relative abundances. Correlations with a BH adjusted *P* value < 0.05 or a significant *P* value at the FDR Q = 1% were considered significant. Each node represents an exometabolite (green) or microbial ASV (purple). Edges, or connections between nodes, indicate the strength of the relationship, with thicker edges indicating a higher correlation coefficient (weight). Edge color represents the direction of the correlation, with positive correlations in red and negative correlations in blue. B) Zoomed in view of the pantothenic acid node and its connected microbial ASVs. C) Zoomed in view of a large sub-network composed of amino acid compounds and their connected microbial ASVs. D) Zoomed in view of the ciliatine node and its connected microbial ASVs.

**Supplemental references:**

1. Widner B, Kido Soule MC, Ferrer-González FX *et al.* Quantification of Amine- and alcohol-containing metabolites in saline samples using pre-extraction benzoyl chloride derivatization and ultrahigh performance liquid chromatography tandem mass spectrometry (UHPLC MS/MS). *Anal Chem* 2021;**93**:4809–17.

2. Garcia BM, Becker CC, Weber L *et al.* Benzoyl chloride derivatization advances the quantification of dissolved polar metabolites on coral reefs. *J Proteome Res* 2024;**23**:2041–53.

3. McLaren MR, Callahan BJ. Silva 138.1 prokaryotic SSU taxonomic training data formatted for DADA2. 2021, DOI: 10.5281/ZENODO.4587955.

4. Oksanen J, Simpson GL, Blanchet FG *et al.* vegan: Community Ecology Package. 2022.

5. Wickham H. ggplot2: Elegant Graphics for Data Analysis. 2016.

6. Gloor GB, Macklaim JM, Pawlowsky-Glahn V *et al.* Microbiome datasets are compositional: And this is not optional. *Front Microbiol* 2017;**8**:2224.

7. Martino C, Morton JT, Marotz CA *et al.* A novel sparse compositional technique reveals microbial perturbations. *mSystems* 2019;**4**:e00016-19.

8. Anderson MJ, Gorley RN, Clarke KR. PERMANOVA+ for PRIMER: Guide to software and statistical methods. *PRIMER-e Learning Hub* 2008.

9. Zhang W (Gordon), Jia Y, Apprill A *et al.* Fine-scale hydrodynamics around St. John, U.S. Virgin Islands. Part I: spatial and temporal heterogeneity in the coastal environment. *Front Mar Sci* 2025;**12**:1464627.

10. Chao A, Chazdon RL, Colwell RK *et al.* Abundance-based similarity indices and their estimation when there are unseen species in samples. *Biometrics* 2006;**62**:361–71.
