# Supplementary figures and images for: Spatiotemporal and hydrodynamic influences on microbial and exometabolite dynamics in coral reef and seagrass ecosystems
Source: ISME J. 2026 Jul 6;20(1):wrag177. doi: 10.1093/ismejo/wrag177 (PMC13403184; doi:10.1093/ismejo/wrag177)

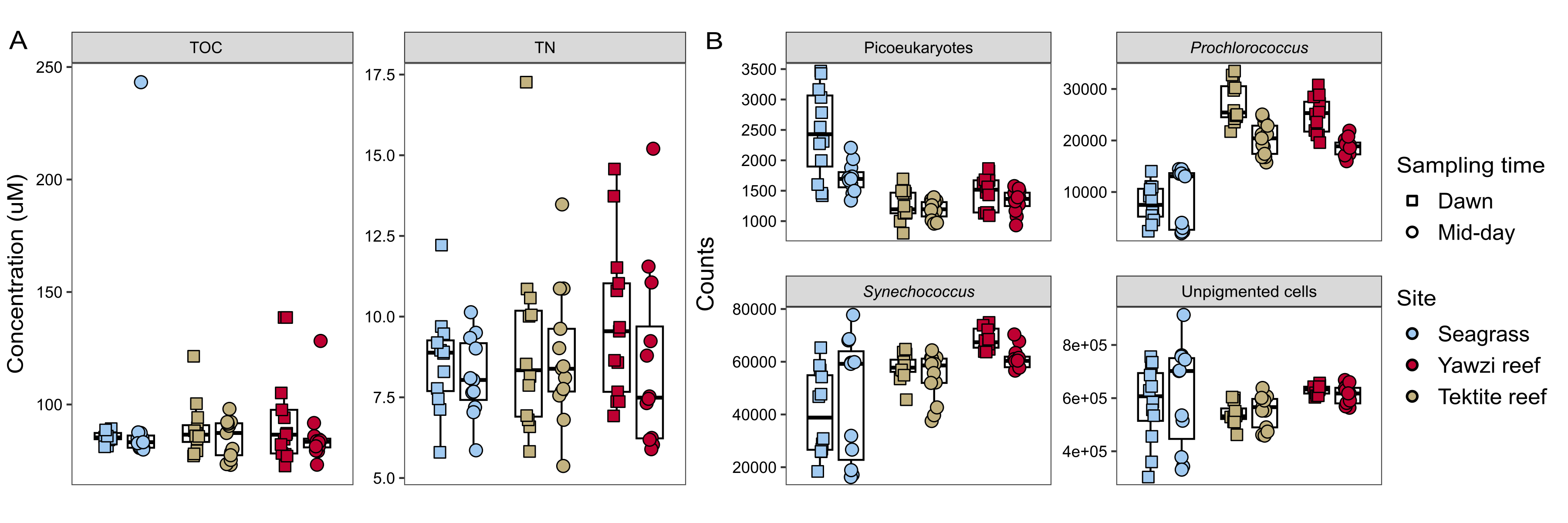

Supplement: Supplementary_material_wrag177 [file supplementary_material_wrag177.zip › FigureS1_WaterChem_FCM_CINARTemporal_20260519.png]

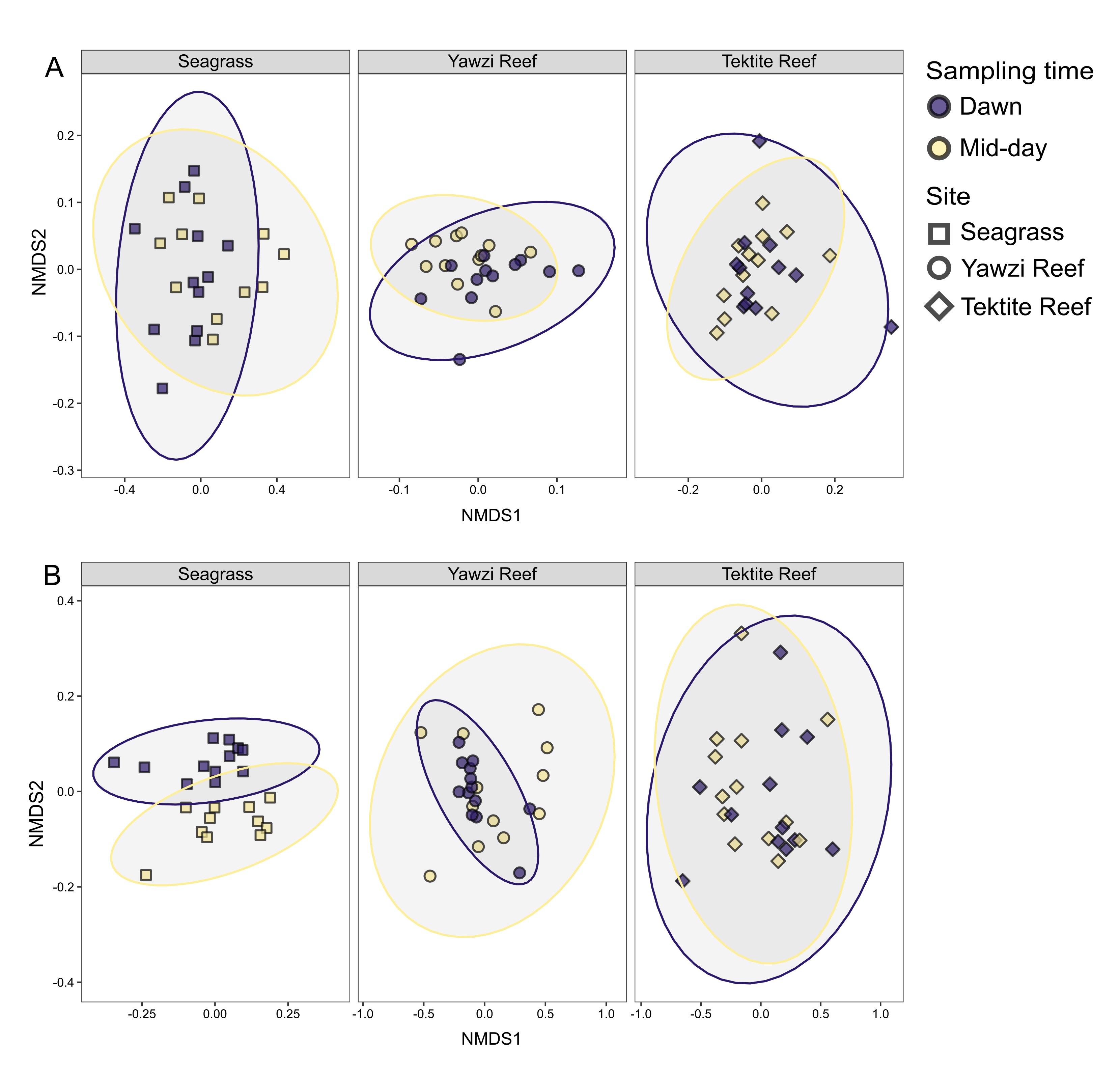

Supplement: Supplementary_material_wrag177 [file supplementary_material_wrag177.zip › FigureS2_SiteSpecific_NMDS_20260519.png]

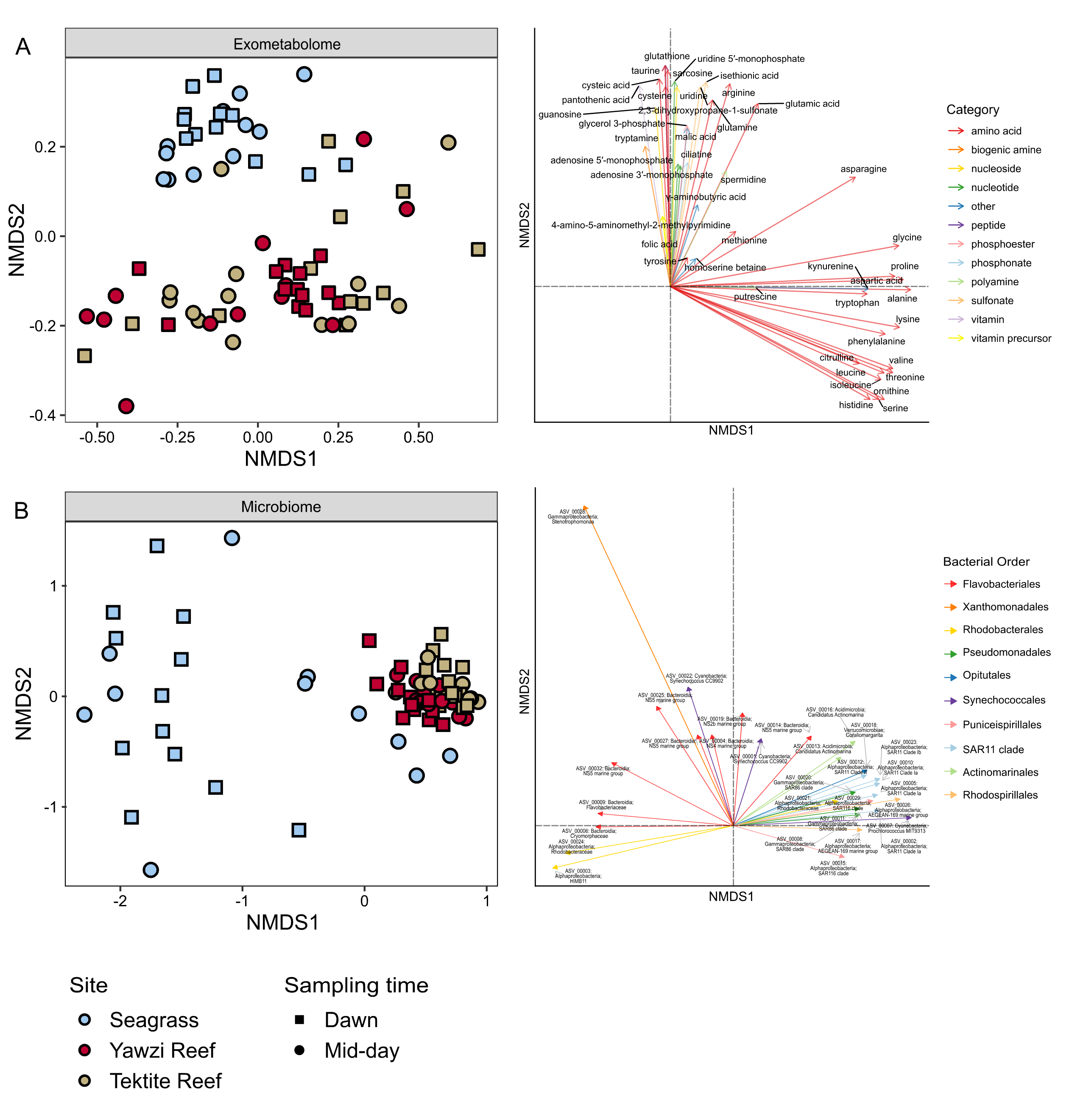

Supplement: Supplementary_material_wrag177 [file supplementary_material_wrag177.zip › FigureS3NEW_NMDS_wVectors_20260512.png]

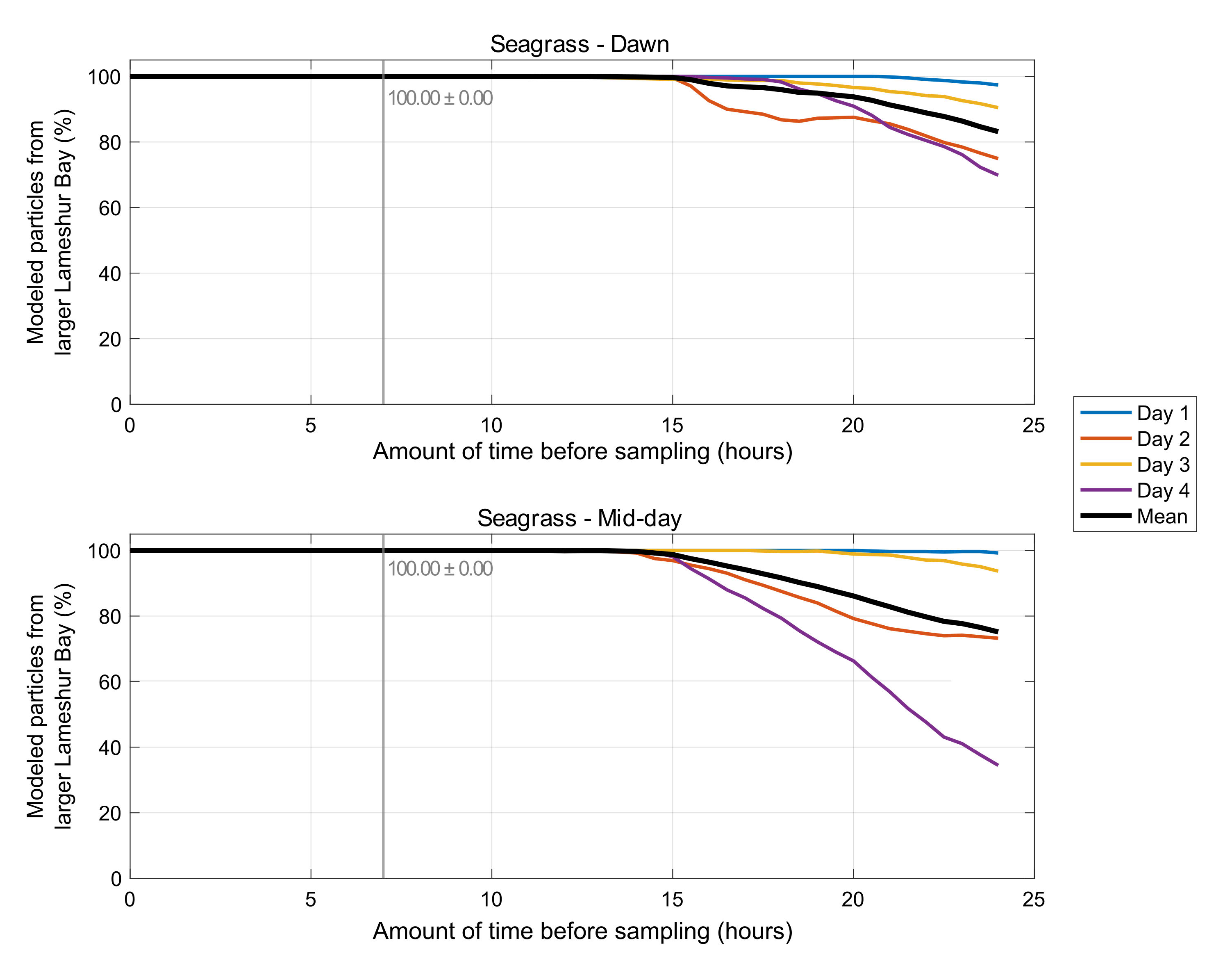

Supplement: Supplementary_material_wrag177 [file supplementary_material_wrag177.zip › FigureS4_Seagrass_PercentCoastalArea_20260519.png]

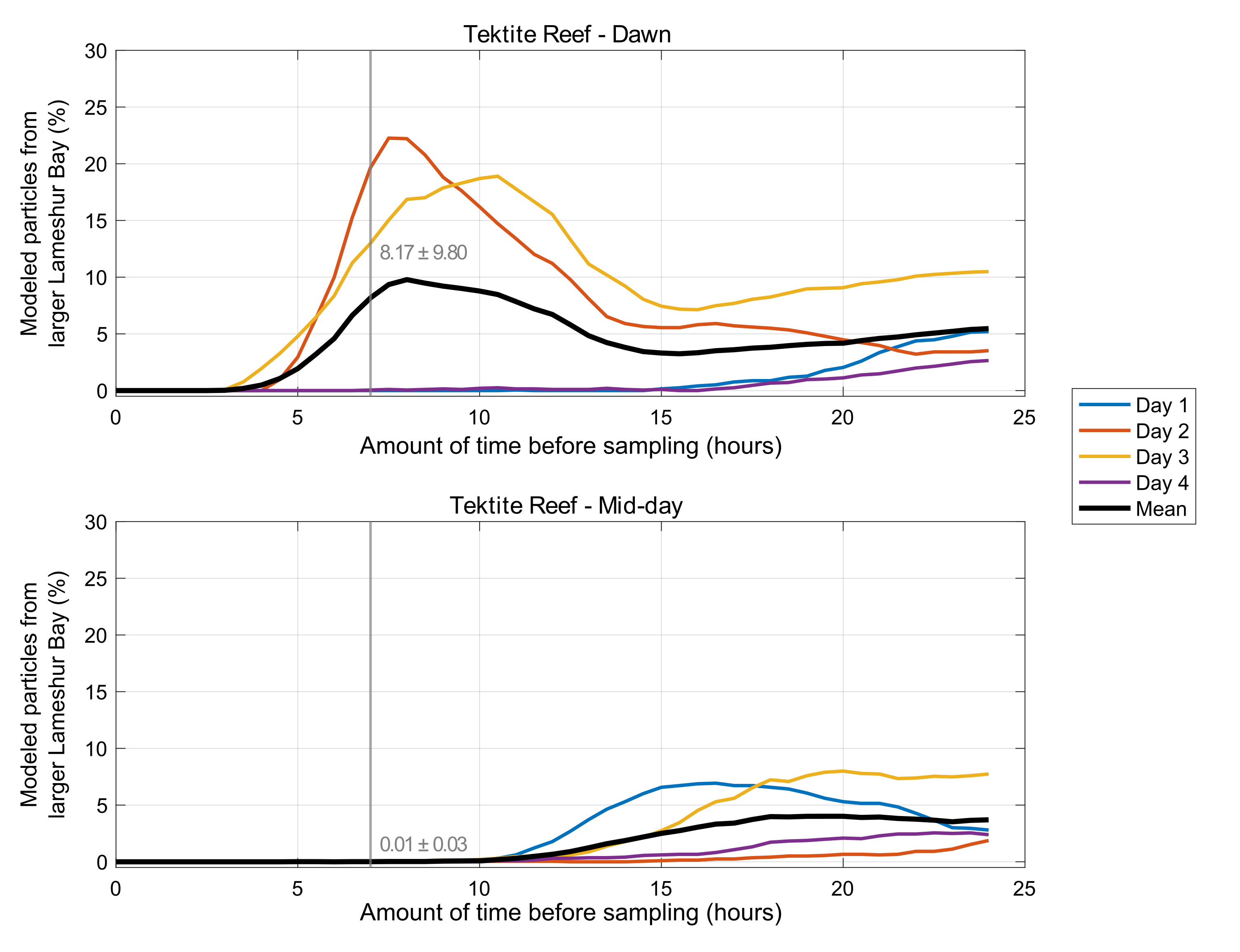

Supplement: Supplementary_material_wrag177 [file supplementary_material_wrag177.zip › FigureS5_Tektite_PercentCoastalArea_20260519.png]

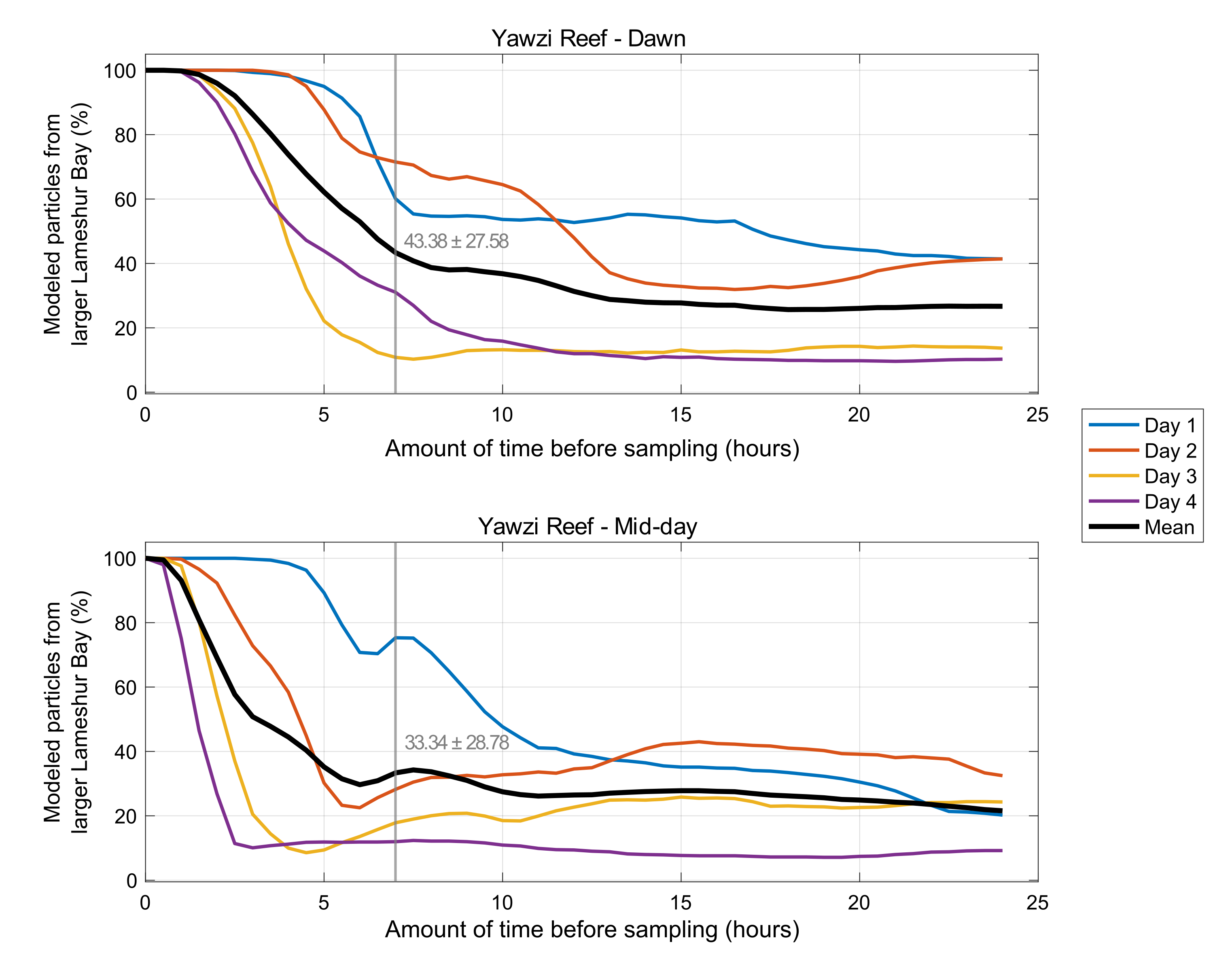

Supplement: Supplementary_material_wrag177 [file supplementary_material_wrag177.zip › FigureS6_Yawzi_PercentCoastalArea_20260519.png]

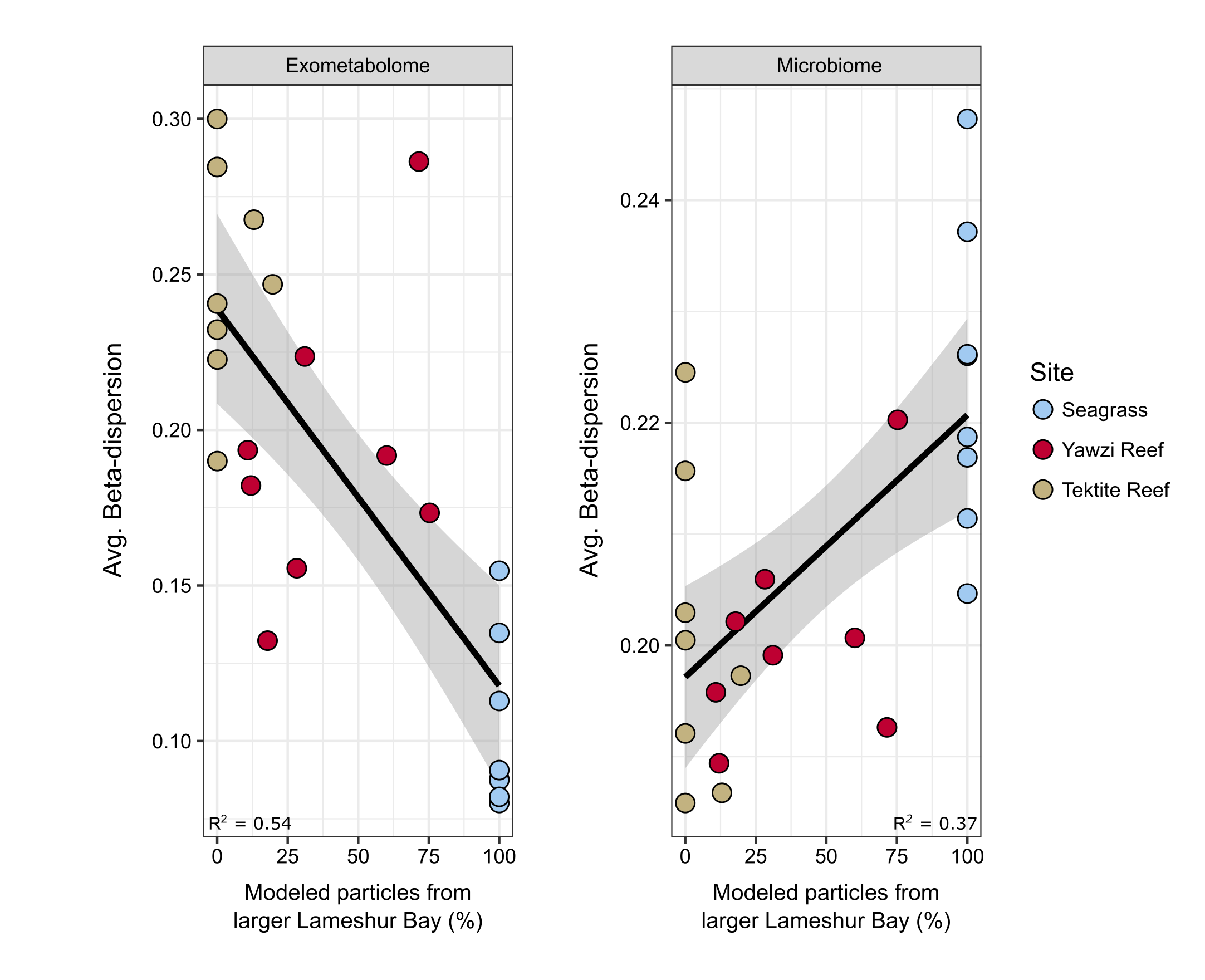

Supplement: Supplementary_material_wrag177 [file supplementary_material_wrag177.zip › FigureS7_LM_HydrodynamicsBetaDispersion_20260519.png]

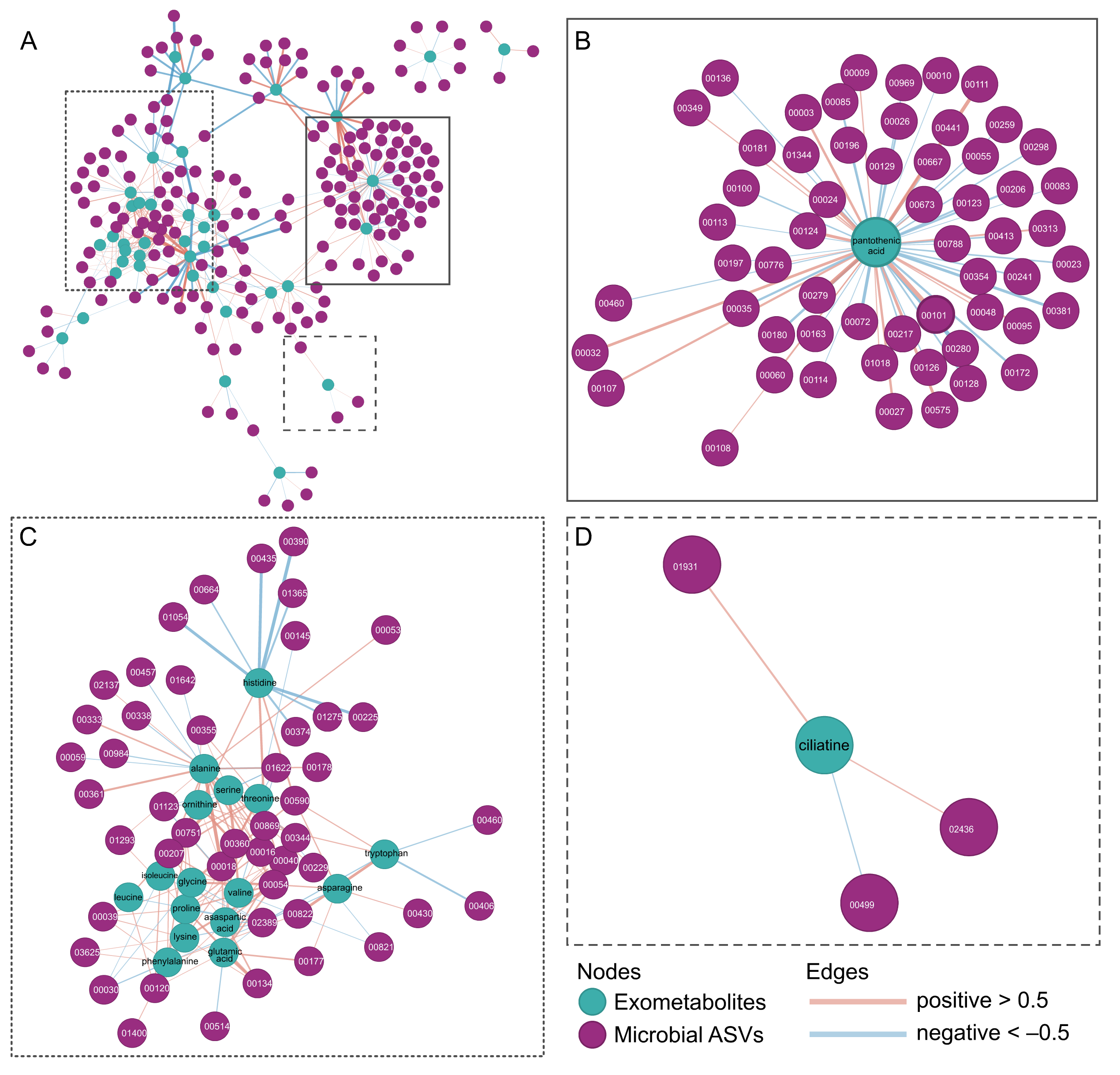

Supplement: Supplementary_material_wrag177 [file supplementary_material_wrag177.zip › FigureS8NEW_YawziReef_CorrNetwork_20260519.png]
